# Gold nanozyme-decorated Cu/Mn MOFs simultaneously enhances immunogenicity and alleviates immunosuppression in triple-negative breast cancer

**DOI:** 10.1186/s12951-026-04482-3

**Published:** 2026-04-25

**Authors:** Haibo Lan, Zede Wu, Jinghua Xia, Qiuyu Li, Mengdan Gao, Zhuoxiu Cai, Weijing Tan, Minyi Liu, Ziting Xu, Yang Gao, Li Zhang, Bingxia Zhao, Yingjia Li, Yu Liang

**Affiliations:** 1 Department of Medicine Ultrasonics, Nanfang Hospital, Southern Medical University, Guangzhou, 510515 China; 2 Cancer Research Institute, School of Basic Medical Sciences, Southern Medical University, Guangzhou, 510515 China; 3 Department of Medical Imaging Center, Nanfang Hospital, Southern Medical University, Guangzhou, 510515 P.R. China; 4 Experiment Education/Administration Center, School of Basic Medical Science, Southern Medical University, Guangzhou, 510515 P.R. China

**Keywords:** Ferroptosis modulation, Nanozyme, Bimetallic MOFs, Tumor microenvironment, Anti-tumor immunity

## Abstract

**Introduction:**

The efficacy of immunotherapy in triple-negative breast cancer (TNBC) is primarily challenged by its inherent low immunogenicity, which is further undermined by the immunosuppressive activity of tumor-associated polymorphonuclear myeloid-derived suppressor cells (PMN-MDSCs). The hypoxic microenvironment renders PMN-MDSCs susceptible to ferroptosis and exacerbates immunosuppression.

**Objectives:**

This study aimed to design a multifunctional nanoplatform comprising Cu/Mn bimetallic metal-organic frameworks decorated with gold (Au) nanozymes (CMAP MOFs) that simultaneously enhances tumor immunogenicity and alleviates immunosuppression.

**Methods:**

The characteristics of the nanosystem were evaluated using transmission electron microscopy, X-ray photoelectron spectroscopy, Fourier transform infrared spectroscopy, and ultraviolet-visible spectrophotometry. In vitro experiments employed Western blotting, immunofluorescence, and flow cytometry to investigate the mechanisms of enhanced immunogenicity and alleviated immunosuppression in 4T1 cells. In vivo experiments evaluated the therapeutic efficacy of the nanosystem in BALB/c mice bearing implanted 4T1 tumors.

**Results:**

Within tumor cells, the CMAP MOFs undergo GSH-responsive disintegration. The released Cu/Mn ions catalyze Fenton-like reaction to induce tumor cell ferroptosis and mitochondrial DNA damage, activating cGAS-STING pathway, and facilitating CD8^+^ T cells recruitment. Simultaneously, released Au nanozymes catalyze H_2_O_2_ conversion to O_2_, alleviating hypoxia and preventing PMN-MDSCs ferroptosis and associated immunosuppression. While the immunogenicity-enhancing CMP MOFs group increased CD8⁺ T cell infiltration from 21% to 28.3% with 74.1% tumor growth inhibition, the combined immunogenicity enhancement and immunosuppression relief CMAP MOFs group further boosted CD8⁺ T cell infiltration to 32.4% with superior tumor growth inhibition of 81.3%, with IFN-γ and GZMB expression elevated by 2.2-fold and 2.0-fold (CMP MOFs) and 3.4-fold and 3.0-fold (CMAP MOFs) compared to control. This strategy offers a promising approach for enhancing immunotherapy in TNBC patients.

**Graphical Abstract:**

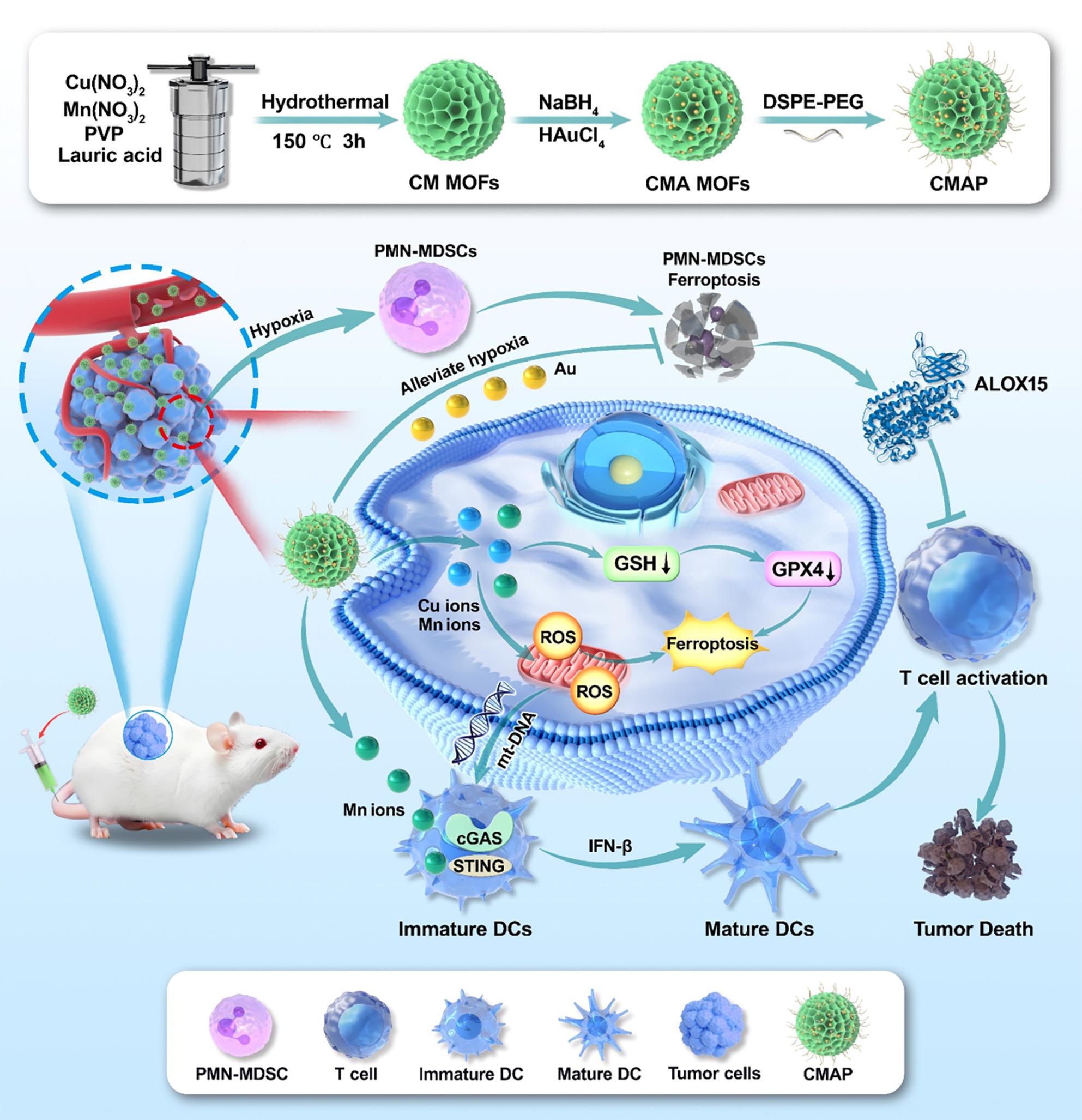

**Supplementary Information:**

The online version contains supplementary material available at 10.1186/s12951-026-04482-3.

## Introduction

 Triple-negative breast cancer (TNBC), characterized by its high heterogeneity, poor prognosis, and elevated risk of recurrence and metastasis, is regarded as one of the most challenging subtypes when compared with other types of breast cancer, making it a stubborn “fortress” in breast cancer research [1–3]. Tumor immunity has been verified to be of great importance to treatment of TNBC, with diverse immune cells participating in the surveillance, recognition, and elimination through different mechanisms [4]. Recent research and clinical trials have shown that TNBC is an immunogenic disease that can be targeted with immunotherapy under the consequence of resistant to chemotherapy and other drugs [5–8].

However, the efficacy of immunotherapy in TNBC remains constrained by two critical barriers: insufficient tumor immunogenicity and the immunosuppressive tumor microenvironment (TME) [9, 10]. The cyclic GMP-AMP synthase-stimulator of interferon genes (cGAS-STING) pathway has emerged as an effective strategy to enhance antitumor immune responses [11]. Notably, current agonist-based approaches often fail to achieve therapeutic-level signaling to elicit clinically meaningful immune responses [12]. Therefore, exploiting new strategies capable of robust STING activation is highly desirable. Ferroptosis, characterized by profound oxidative stress and mitochondrial damage, represents a promising approach against therapy-resistant cancers [13–18]. Crucially, oxidative mitochondrial damage triggers cytosolic oxidized mitochondrial DNA (ox-mtDNA) release, serving as a potent STING activation mechanism [19–21]. Remarkably, Mn²⁺ has been demonstrated to enhance cGAS sensitivity to DNA by tens of thousands of times and strengthen the binding of STING to cGAMP by hundreds of times, thereby inducing a hyperactivated immune state in cells [22]. Phase 1 clinical studies revealed that the use of manganese ions in conjunction with PD-1 inhibitors achieved an objective response rate of 45.5% and a disease control rate of 90.9%, with satisfactory clinical safety [23]. Currently, Phase 2 clinical trials are being conducted in an orderly manner. Furthermore, Cu/Mn-based nanoagents have demonstrated the ability to simultaneously induce tumor cell ferroptosis while activating the cGAS-STING pathway [24], offering a promising dual-functional approach to enhance tumor immunogenicity in TNBC [25].

In addition to enhancing tumor immunogenicity, counteracting the immunosuppressive tumor microenvironment also constitutes a major challenge for TNBC immunotherapy. Tumor pathologically activated neutrophils (PMN-MDSCs), also termed polymorphonuclear myeloid-derived suppressor cells (PMN-MDSCs), are major negative regulators of anti-tumor immunity [26–29]. Recent studies reveal that PMN-MDSCs within the tumor hypoxic microenvironment, but not those derived from bone marrow or spleen, exhibit heightened susceptibility to ferroptosis. Mechanistically, hypoxia induces downregulation of Glutathione Peroxidase 4 (GPX4) in tumor-infiltrating PMN-MDSCs, thereby promoting their ferroptosis. These ferroptotic PMN-MDSCs release immunosuppressive mediators that potently inhibit T cell functionality, thereby hindering effective immunotherapy [30]. Alleviating TME hypoxia could mitigate ferroptosis in PMN-MDSCs, thereby ameliorating the immunosuppressive microenvironment. Gold (Au) nanozymes have garnered significant attention for their potential to address the hypoxic TME due to their unique catalase-like activity [31, 32]. Incorporating Au nanozymes provides a simple approach for in situ oxygen production and holds great promise for immunosuppression relief in TNBC.

Herein, we aim to simultaneously enhance tumor immunogenicity and alleviate immunosuppression for enhanced TNBC treatment. Our strategy involves: (1) inducing ferroptosis in cancer cells and activating the STING pathway to enhance immunogenicity, and (2) leveraging the differential sensitivity of PMN-MDSCs to ferroptosis under hypoxic versus normoxic conditions to reduce their ferroptosis susceptibility and reverse their immunosuppression. Accordingly, Cu/Mn bimetallic metal-organic frameworks (CM MOFs) decorated with Au nanozymes (CMA MOFs) and PEGylation (CMAP MOFs) were designed. Triggered by high intracellular GSH levels in tumor cells, CMAP MOFs gradually disintegrated, releasing Au nanozymes and Cu/Mn ions. The released Cu/Mn ions catalyze Fenton-like reactions to generate toxic ROS, triggering tumor cell ferroptosis and ox-mtDNA release, while Mn²⁺ ions serve as powerful amplifiers, significantly enhancing cGAS-STING pathway activation, promoting the maturation of DCs and facilitating the recruitment of CD8^+^T cells. Meanwhile, Au nanozymes efficiently alleviate tumor hypoxia, thereby protecting PMN-MDSCs from ferroptosis and mitigating their immunosuppressive effects on T cell functionality. By synergistically enhancing tumor immunogenicity via cGAS-STING activation and alleviating PMN-MDSCs ferroptosis-induced immunosuppression, our CMAP MOFs platform demonstrates a promising therapeutic strategy for addressing the significant clinical challenges in TNBC treatment and enhancing immunotherapy responses in patients with poor prognosis.

**Scheme 1:**
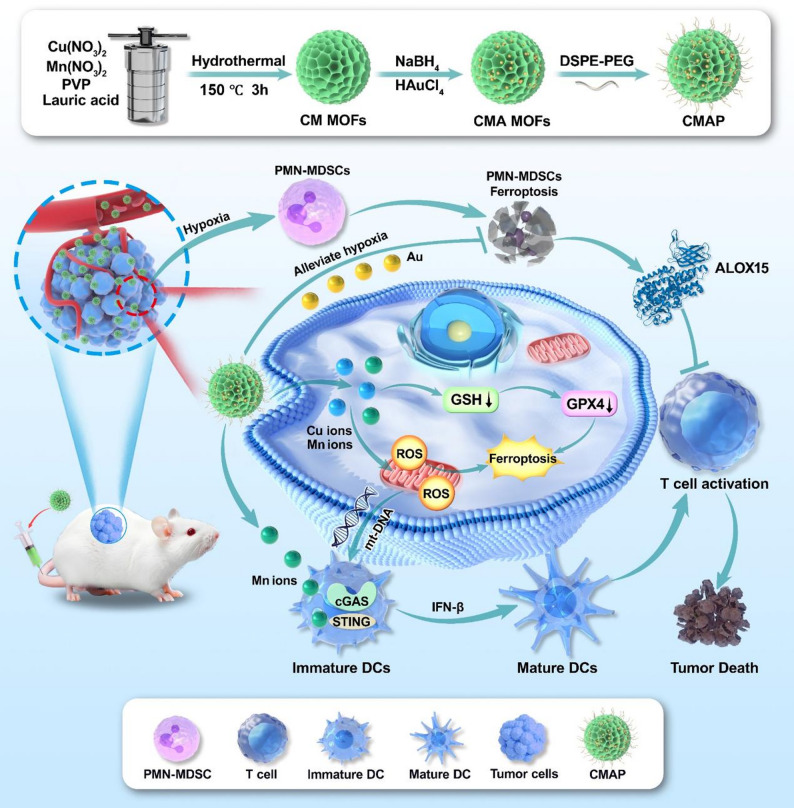
Schematic illustration of CMAP MOFs for simultaneous tumor immunogenicity enhancement and immunosuppression alleviation in TNBC treatment

## Materials and methods

*Materials and reagents* Methanol, DSPE-mPEG (MW = 2000), Copper nitrate trihydrate (Cu(NO_3_)_2_·3H_2_O), NaBH_4_, Dithiodiglycolic acid and Lauric acid, were sourced from Macklin (Shanghai, China). Manganese (II)nitrate tetrahydrate (Mn(NO_3_)_2_·4H_2_O) and HAuCl_4_ were from Aladdin, Shanghai, China. The polyvinylpyrrolidone utilized in this study was procured from Solarbio, a prominent supplier located in Beijing, China. No further purification was performed on the remaining chemical reagents before use.

*Synthesis of CMAP MOFs complexes* The CM MOFs were synthesized through hydrothermal process. Briefly, 0.159 mmol Mn(NO_3_)_2_·4H_2_O, 0.107 mmol Cu(NO_3_)_2_·3H_2_O, 100 mg dithiodiglycolic acid, 75 mg PVP, and 500 mg Lauric acid were added into 25 mL Methanol solution under vigorous stirring. Following a 10-minute interval, the reaction mixture was loaded into a Teflon-lined autoclave and subjected to a thermal treatment at 150 ℃ for a duration of 3 h. The precipitates of CM MOFs were obtained by centrifugation and then thoroughly washed with ethanol in three successive cycles.

In a 30 mL glass bottle, 10 mL of a 1 mg/mL CM MOFs ethanol solution was subjected to ultrasound treatment. Then, 60 µL of a 50 mg/mL HAuCl_4_ solution was introduced and mixed vigorously for a period of 5 min. Next, 1 mL of a freshly prepared 10 mg/mL NaBH_4_ solution was added dropwise and then subjected to stirring for a duration of 15 min. The mixture was washed three times with ethanol to yield the CMA MOFs dispersion in ethanol.

Then, a total of 30 mg DSPE-mPEG was added and stirred overnight. The surface modified CMAP MOFs complexes were purified by centrifugation. The complexes, once prepared, were resuspended in PBS and maintained at a temperature of 4 ℃.

*Characterization of MOFs* Elemental mapping was performed using high-angle annular dark-field scanning transmission electron microscopy (HAADF-STEM) and conventional transmission electron microscopy (TEM). The analyses were carried out with a JEOL JEM-2100 F TEM instrument operated at an accelerating voltage of 200 kV. The UVeVis absorption spectra was noted by a Shimadzu UV-2600 UVeVis spectrophotometer. Fourier transform infrared spectroscopy (FTIR) analysis was conducted using a Thermo Fisher Nicolet 6700 instrument. The valence states of Cu, Mn, and Au within the CMAP MOFs were investigated through X-ray photoelectron spectroscopy (XPS) using an ESCALAB 250 spectrometer.

*In vitro TME-activated experiment* The structural degradation of CMAP MOFs triggered by GSH was examined by exposing CMAP MOFs solutions to PBS, in the presence or absence of 10 mmol/L GSH. The structures were periodically observed using TEM (JEOL) at predetermined time points.

Since MB (MB, Macklin, China) can be transformed into colorless by ·OH, MB were employed to investigate the ·OH generation potential of CMAP MOFs. The degradation of MB after incubation with CMAP MOFs under different conditions was noted at 665 nm, the notable absorption peak of MB. Inductively coupled plasma optical emission spectroscopy (ICP-OES) was employed to determine the elemental concentration of MOFs after incubation with 2 mM GSH.

The concentration of dissolved oxygen (O_2_) in the aqueous solution was determined using a DO200 portable dissolved oxygen meter, manufactured by Clean Instruments, Co., in the United States. A 50 mL centrifuge tube filled with nitrogen was sealed with a rubber plug, followed by the addition of 10 mL deoxygenated water (1 mmol/L H_2_O_2_). The dynamic fluctuations in O_2_ concentration were tracked in real-time by immersing the electrode probe of the O_2_ meter into the tube. Subsequently, a 5 mL volume of the sample solution, with a concentration of 1 mg/mL, was injected into the system. O_2_ concentrations were recorded every 30 s for a total duration of 10 min.

*Cell culture studies* The 4T1 and NIH/3T3 cell lines were sourced from the Research Center of Clinical Medicine at Nanfang Hospital, located in Guangzhou, China. Cells were cultured in Roswell Park Memorial Institute 1640 medium (RPMI 1640) containing 10% (vol/vol) fetal bovine serum (FBS) and 1% (vol/vol) penicillin–streptomycin solution under a 5% CO_2_ atmosphere at 37 °C.

*Cell viability assays* For the measurement of cell viability, cells were plated in 96-well plates at densities varying between 5,000 and 10,000 cells per well. After the cells were firmly attached to the plates, different treatments were initiated. Once the cells had been treated for the designated time, 100 µL of fresh medium with 10% CCK-8 reagent (Biosharp, Canada) was introduced to replace the medium in each well. For another 1 h incubation, a microplate reader, from BioTek, USA, was used to detect every plate at an absorbance of 450 nm. The viability of cells was determined by the equation: cell viability (%) = ((absorbance of tested compound – absorbance of blank) ÷ (absorbance of control – absorbance of blank)) × 100.

*Measurement of ROS and lipid peroxidation* To measure ROS and lipid peroxidation, H2DCFDA (Thermo Fisher Scientific, D399) was used as a ROS-sensitive probe, while BODIPY 581/591 C11, produced by Thermo Fisher Scientific, was employed as a lipid peroxidation sensor. Briefly, cells in confocal dish and 6-well plates were labeled using 1 µM H2DCFDA dye or 1 µM BODIPY 581/591 C11 dye for a duration of 30 min after various treatments. The cells were observed and pictured by a fluorescent microscope (DS-RI2, Nikon). At the same time, cells were collected, rinsed with PBS, and prepared for flow cytometry to measure and analyze ROS or lipid peroxidation levels.

*Evaluation of mitochondrial function* 4T1 cells were pre-treated with PBS, CMP MOFs, CMAP MOFs. Following a period of 24 h, the treated cells were collected for dectection of mitochondrial function with a JC-1 kit (C2003S, Beyoime, China). Briefly, 1 mL of JC-1 staining solution was added to the cells and mixed to ensure homogeneity. Following a 20-minute incubation at 37 °C in a cell culture incubator, the cells were washed twice using JC-1 staining buffer. Fluorescent microscopy (DS-RI2, Nikon) was employed to observe and capture images of the cells. At the same time, cells were collected, rinsed with PBS, and then processed for flow cytometry analysis to assess JC-1 accumulation.

*The detection of oxidative DNA damage and mitochondria* Initially, the 4T1 cells were harvested and planted in confocal dish. Once the cells had attached, they were treated with PBS, CMP MOFs, and CMAP MOFs, respectively. After the treatment, cells were fixed with 4% paraformaldehyde for a duration of 10 min, followed by permeabilization in a 0.3% Triton X-100 solution for an additional 10 min. Subsequently, both 8-OHdG antibody (1:200; Novus Biologicals) and TOMM20 antibody (1:200; Abcam) were applied for incubationg with the treated cells. After a duration of 12 h, the treated cells were successively maintained with secondary antibody for 1 h, and with DAPI for a duration of 10 min. Ultimately, the mitochondrial and Oxidized mitochondrial DNA (Ox-mtDNA) were assessed by immunofluorescence.

*Bone marrow-derived dendritic cells (BMDCs) maturation in vitro* Bone marrow-derived dendritic cells (BMDCs) were isolated from the femurs and tibias of 6–8 weeks old female C57BL/6 mice, the cells were cultured in RPMI 1640 medium supplemented with 10% fetal bovine serum (FBS) and 20 ng/mL recombinant murine GM-CSF for 7 days. To evaluate the immunogenic activity in vitro, the supernatant of cells from tumor was collected through the previous described treatment procedure and applied to treat BMDCs for 24 h. Afterward, the treated BMDCs were stained with anti-CD11c(17-0114-82), anti-CD80(11-0801-82) and anti-CD86 (12-0862-82) antibodies (Invitrogen, California, USA). The maturation status of BMDCs was assessed using flow cytometry.

*In vivo tumor targeting* In this study, female BALB/c mice, aged between 4 and 6 weeks, were sourced from Bestest, located in Zhuhai, China. All animal experimental procedures adhered to the institutional guidelines for the care and use of laboratory animals. The conduct of all experiments about animal was subject to rigorous review and formal approval by the Institutional Animal Care and Use Committee of Southern Medical University, with the assigned approval number SMUL2023111. The 4T1 tumor-bearing mice model was created by injecting 5 × 10^6^ cells, suspended in 100 µL of PBS, subcutaneously into the right flank of the mice. Afterwards, the mice with 4T1 tumor-bearing were injected with IR780 loaded CMAP MOFs through intravenous injections in the tail. At the predetermined time points of 0,1, 3, 6, 12, 24 h and 48 h, fluorescence images were captured using a fluorescence imaging system provided by Digital Precision Medicine Company, located in Beijing, China. In order to evaluate the in vivo metabolic profile of CMAP MOFs, the healthy mice were intravenously injected with CMAP MOFs. At the indicated time point (1 h, 6 h, 12 h, 24 h, 48 h), the main organs and tumors were collected, weighted and dissolved in digesting nitric acid. The concentrations of Cu in different samples were measured byinductively coupled plasma mass spectrometry (ICP-MS).

*In vivo cancer treatment* Mice bearing 4T1 tumors were established through xenografting and subsequently allocated into three distinct groups, each comprising five mice: BLANK, CMP MOFs and CMAP MOFs. The dimensions of the tumors were assessed using a digital caliper to monitor their length and width. Subsequently, the anti-tumor efficacy was evaluated by determining the tumor volumes using the formula: volumes (V) = Length × Width^2^ /2. Relative tumor volume was defined as V/V_0_ × 100%, with V₀ representing the tumor volume prior to treatment. The body weights of the mice were assessed at 3-day intervals following the treatment.

*Blood biochemical and Red Blood Cells Hemolysis* Following the intravenous administration of different treatments into BALB/c mice, blood samples (approximately 1000 µL) were obtained and sent to Wuhan Saville Biotechnology Co., Ltd. for comprehensive blood biochemical and hematological analyses, focusing on parameters such as creatine kinase-MB (CK-MB), glutamic-pyruvic transaminase (ALT) and creatinine (CR). The biocompatibility of CMAP MOFs with blood was evaluated using fresh erythrocyte suspensions. Specifically, various concentration of CMAP MOFs were incubated in 1 mL of PBS with 200 µL erythrocyte suspension at 37 °C for 4 h. After the mixture was centrifuged for supernatant collection, the degree of hemolysis was assessed by measuring the UV − vis absorbance of the supernatant.

*Histology and immunohistochemistry* Tumor samples from mouse xenograft models, along with major tissues from each experimental group, were harvested, fixed, and processed for embedding. The samples were subsequently processed for histological examination, employing haematoxylin and eosin (H&E) staining. Concurrently, the tumor samples were processed with overnight incubation at the temperature of 4 °C with a panel of primary antibodies for immunohistochemical analysis. These antibodies included Ki67 (1:500; Cell Signaling Technology, 9661s), GPX4 (1:400; Abcam, ab125066), PTGS2 (1:400; Abcam, ab46545), HIF-1α (1:400; Abcam, ab308433), as well as antibodies targeting CD45, CD11b, Ly6G, CD11c, CD3, and CD8 (1:500; Cell Signaling Technology). Next, the tumor samples were immersed in secondary antibody (ab6728, Abcam). Lastly, photographs were conducted by an inverted fluorescence microscope (DS-RI2, Nikon).

*Western blot analysis and Enzyme-linked immunosorbent assay (ELISA) test* To validate the activation of cGAS-STING pathway and ferroptosis, protein was extracted from tumor tissues or cells. The levels of STING (13647S), phosphorylated STING (p-STING, 72971S), IRF3 (4302S), and phosphorylated IRF3 (p-IRF3, 29047S) (Cell Signaling Technology, Massachusetts, USA), as well as GPX4 (ab125066, Abcam) and PTGS2 (ab179800, Abcam), ALOX15 (ab244205, Abcam) were examined through Western blot analysis. In detail, the tumor cells were harvested and rinsed with PBS. Subsequently, RIPA lysis buffer (Abcells, China) supplemented with phosphatase and protease inhibitors (FDbio, China) was introduced and incubated on ice for 30 min. Following centrifugation at 12,000 rpm for 15 min to extract the proteins, the concentration of the extracted proteins was determined using a BCA protein assay kit. The proteins were denatured by heating in the loading buffer under the circumstance of 100 °C for 5 min. The proteins were subjected to SDS-PAGE electrophoresis using a Bio-Rad mini apparatus, transferred onto PVDF membranes, and incubated with primary antibodies overnight at 4 °C on a shaker. Next, the PVDF membranes were rinsed three times and then exposed in the presence of horseradish peroxidase (HRP)-conjugated secondary antibodies for 1 h. The Western blot images were captured using the Bio-Rad ChemiDoc Touch system (Bio-Rad, USA) after adding 400 µL of ECL chemiluminescent reagent (FDbio, China) directly onto the membrane surface. Bio-Rad Image Lab™ Touch Software was applied to analyze the intensity of the bands. To determine the concentrations of TNF-α, IL-6, IFN-β, and IFN-γ in serum, blood samples collected from mice were allowed to clot at room temperature for 2 h, then centrifuged at 3,000 rpm for 15 min to separate the serum. The resulting supernatant was used for subsequent ELISA analysis, utilizing kits from Jiangsu Meimian Industrial (China).

*Ferroptosis of tumor pathologically activated neutrophils (PMN-MDSCs) in spleens and tumors of mouse* Mice with tumor-bearing were sacrificed after three weeks of 4T1 cells inoculation for the collection of spleens and tumors to be prepared as single-cell suspensions. Fluorochrome-conjugated antibodies of CD45-V450(48-0451-82), CD11b-PE (12-0112-82), Ly6G-ethanoAPC (17-9668-82) and CD71-FITC (11-0711-82) (Invitrogen, California, USA) were applied to stain the cells derived from both spleens and tumors about 30 min at 4 °C for fluorescent labeling of PMN-MDSCs ferroptosis. The cells from spleens and tumors were then washed three times in PBS for PMN-MDSCs ferroptosis analysis by flow cytometry.

*Isolation of mouse cells Single-cell* suspensions were prepared from spleen and tumor followed by red blood cell removal using ammonium chloride lysis buffer. Tumor tissues were processed using the Mouse Tumor Dissociation Kit according to the manufacturer’s recommendations (Miltenyi). Tumor PMN-MDSCs (CD45+CD11b+Ly6G+) were sorted using the FACS Aria (BD Biosciences) system. PMN-MDSCs were purified using anti-Ly6G microbeads (Miltenyi Biotec) according to the manufacturer’s instructions or sorted using the FACS Aria (CD45+CD11b+Ly6G+) system.

*Alleviating immunosuppression by hypoxia alleviation in vivo* To explore the effects of hypoxia alleviation on the generation of oxidized phospholipids from PMN-MDSCs ferroptosis. The PMN-MDSCs isolated from tumor tissues following various treatments were administered to verify the level of proteins about hypoxia and ferroptosis, the concentration of the total protein extracted from PMN-MDSCs was quantified by a BCA assay kit. Following separation via 10% SDS-PAGE gel electrophoresis and subsequent transfer to polyvinylidene difluoride (PVDF) membranes, the proteins were subjected to incubation with primary antibodies against GPX4 (ab125066, Abcam), HIF-1α (ab308433, Abcam), ALOX15 (ab244205, Abcam). Ultimately, following incubation with HRP-conjugated antibodies for 1 h, the proteins were then visualized using an ECL kit.

Upon completion of the treatment regimen, the alterations in the abundance of PMN-MDSCs at the tumor site were evaluated via flow cytometry. Typically, the mice were euthanized for the collection of their tumors and spleens to create single-cell suspensions. To assess PMN-MDSCs ferroptosis, the suspension of cells was stained with antibodies of anti-mouse CD45-V450, CD11b-PE, Ly6G-APC, and CD71-FITC (Invitrogen, California, USA).

*Activation of cGAS-STING immune pathway in vivo* To assess T-cell subpopulations, suspensions of cell were exposed to antibodies against mouse CD45-V450, CD3-PE/Cy7, and CD8-FITC (Invitrogen, California, USA). For the detection of mature BMDCs, cell suspensions were stained with antibodies targeting mouse CD45-V450, CD11c-PE/Cy7, CD80-FITC, and CD86-PE (Invitrogen, California, USA).

*Statistics analysis* The two-tailed Student’s t-test was employed for comparisons between two groups, whereas one-way analysis of variance (ANOVA) was used for comparisons among multiple groups. A p value less than 0.05 was deemed to signify a significant difference. The significance levels were denoted as follows: *p < 0.05, **p < 0.01, ***p < 0.001, ****p < 0.0001. Quantitative results are reported as mean ± standard deviation (SD). The bar charts and curve plots were generated using GraphPad Prism version 9 software.

## Results and discussion

### Synthesis and characterization of CMAP MOFs

**Fig. 1:**
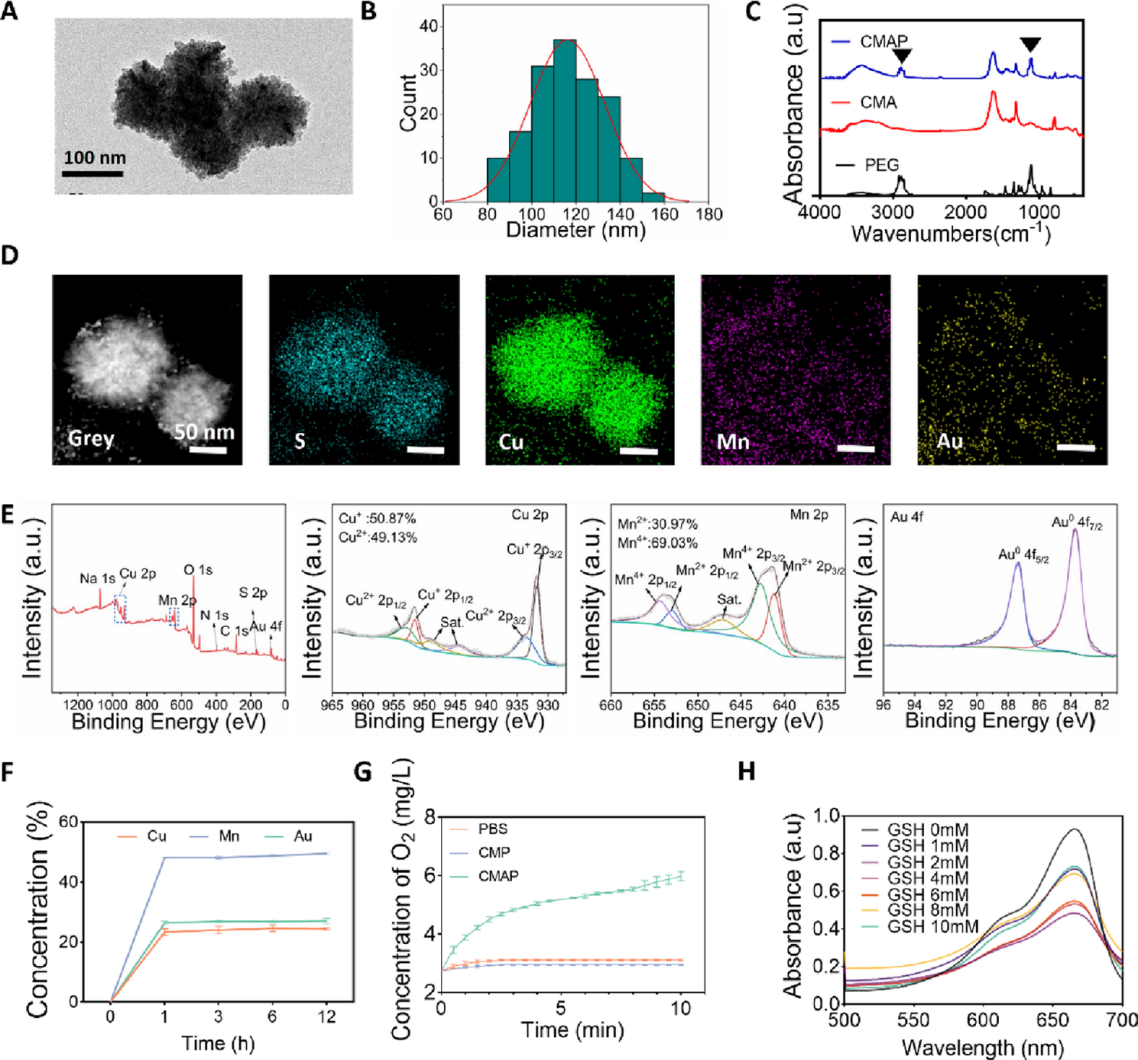
Characterization of CMAP MOFs. (**A**) TEM images of CMAP MOFs. Scale bar: 100 nm. (**B**) Particle size distribution of CMAP MOFs. (**C**) FTIR of PEG, CMA MOFs and CMAP MOFs. (**D**) STEM-EDS elemental maps of CMAP MOFs. Scale bar: 50 nm. (**E**) High-resolution XPS spectra of CMA MOFs. (**F**) Detection of ion releasing from CMAP MOFs after incubation with GSH (2 mM) by ICP. Data were presented as mean ± SD (*n* = 3 for each group). (**G**) The generated O_2_ concentration in different MOFs at predetermined time points. Data were presented as mean ± SD (*n* = 3 for each group). (**H**) The UV-vis absorption spectra of MB degradation in CMAP MOFs solutions with the existence of H_2_O_2_ (10 mM) and different concentration of GSH

The development and construction of CMAP MOFs are illustrated in Scheme 1. Cu/Mn MOFs (CM MOFs) were synthesized using a hydrothermal method according to previous report with modification [33]. Gold (Au) nanozymes were subsequently decorated in situ onto the surface of the as-prepared MOFs to yield CMA MOFs. To improve the biocompatibility and prolong the blood circulation, CMA MOFs was modified with DSPE-mPEG (CMAP MOFs). Figure 1A presents TEM images of CMAP MOFs, indicating a uniform spherical morphology. The average diameter of CMAP MOFs was determined to be 116.2 ± 16.86 nm, with a surface potential of -33 mV (Fig. 1B & S1). Fourier transform infrared spectra (FTIR) revealed that the peaks of 3000 –2800 cm^− 1^ and 1100–1150 cm^− 1^ (connected to methyl groups symmetric stretching vibrations and C-O-C stretching vibrations in PEG) were both observed at PEG and CMAP MOFs, which were absent in CMA MOFs, confirming successful PEGylation of CMA MOFs (Fig. 1C). High-angle annular dark-field-scanning transmission electron microscopy-based element mapping images verified the uniform distribution of Cu, Mn, and Au, demonstrating the successful incorporation of Au nanozymes, forming CMAP MOFs (Fig. 1D). The incorporation of Mn, Cu, and Au in CMAP MOFs was further confirmed through X-ray photoelectron spectroscopy (XPS) (Fig. 1E).

Given that CMAP MOFs contain disulfide bonds, their redox-responsive behavior was comprehensively investigated. In a simulated microenvironment (PBS containing 10 mM GSH), CMAP MOFs underwent progressive structural collapse over time (Fig. S2). This disintegration facilitated the Cu, Mn dual ions release for toxic ⋅OH generation *via* Fenton-like reaction, and enabled Au nanozymes to catalyzing the intratumoral conversion of excess H_2_O_2_ into oxygen (O_2_). ICP analysis revealed a continuous and time-dependent release of Cu, Mn, and Au in the presence of 2 mM GSH (Fig. 1F). The oxygen-generating capability of the decorated Au nanozymes was assessed in a solution containing a high concentration of H₂O₂ using a dissolved oxygen meter. Compared to the PBS and CMP MOFs groups, CMAP MOFs rapidly elevated dissolved oxygen levels, exceeding 6 mg/L within ten minutes (Fig. 1G), demonstrating that CMAP MOFs possess excellent oxygen-generating ability for tumor hypoxia alleviation. To evaluate GSH-responsive ⋅OH generating efficacy of CMAP MOFs, methylene blue (MB) was applied as an indicator. The relationship between GSH concentration and ⋅OH generation was non-linear. MB absorption decreased with increasing GSH concentration up to 2 mM, but rebounded at higher concentrations as excessive GSH scavenges a portion of the newly generated ⋅OH (Fig. 1H & S3). To assess their stability in blood, the CMAP MOFs were incubated with PBS, FBS, and RPMI 1640 medium at 37 °C for 0, 12, 24, and 48 h. During this period, both particle size and the particle size distribution index (PDI) remained stable, with no significant aggregation or degradation observed, indicating good colloidal stability under physiological conditions (Fig. S4).

### GSH-responsive ferroptosis induction in tumor cells

**Fig. 2:**
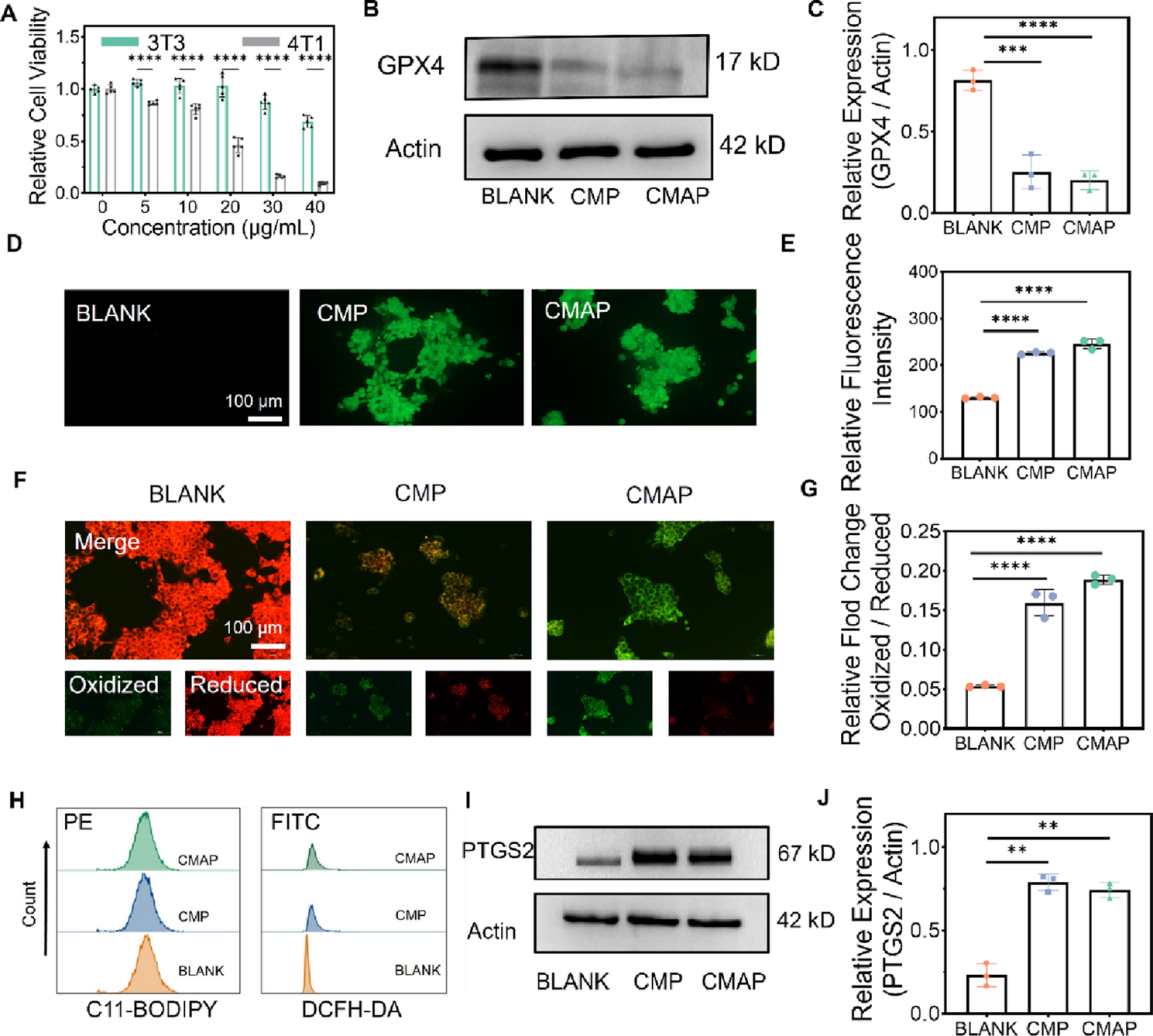
Tumor-targeted ferroptosis induction of MOFs in vitro. (**A**) Cell viabilities of 4T1 and NIH/3T3 cells after incubation with CMAP MOFs at different concentrations. (**B**) Western blotting analysis of GPX4 expression in 4T1 cells treated with different groups (**C**) and relative quantification. (**D**) DCFH-DA assay of 4T1 cells treated with different groups, scale bar=100 μm. (**E**) Corresponding flow cytometry analyses of DCFH-DA assay of 4T1 cells treated with different groups. (**F**) Fluorescence images of 4T1 cells treated with different groups, C11-BODIPY was used to assess lipid peroxidation. Scale bars༝100 μm. (**G**) (**H**) Flow cytometry analyses of lipid peroxidation in 4T1 cells treated with different groups, C11-BODIPY was used to assess lipid peroxidation and Relative fold changes of lipid peroxidation in Oxidized/Reduced. (PE channel represents the C11-BODIPY signal, and the FITC channel represents the DCFH-DA signal). Date are presented as mean ± SD (n=3). (**I**) Western blotting analysis of PTGS2 expression in 4T1 cells treated with different groups, (**J**) and relative quantification. Data are presented as mean ± SD (*n* = 3). **p < 0.01, ***p < 0.001, ****p < 0.0001

Cellular uptake studies using RhB-labeled MOFs showed significantly enhanced red fluorescence intensity in CMP MOFs and CMAP MOFs treated 4T1 cells compared to the BLANK group, indicating efficient internalization (Fig. S5). In vitro cytotoxicity assessed by CCK-8 assay revealed that NIH/3T3 cells maintained a viability of over 70% after 24 h exposure to 40 µg/mL CMAP MOFs, whereas the viability of 4T1 cells decreased significantly under the same treatments (Fig. 2A). This selective cytotoxicity indicates their potential as specific antitumor agents by exploiting the high concentration of GSH and H_2_O_2_ in cancer cell.

The disulfide bonds in as-synthesized CMP MOFs and CMAP MOFs could effectively deplete intracellular GSH in cancer cells. Subsequently, these bonds were cleaved, leading to structure disintegration and the release of Cu/Mn ions and Au nanozymes. Critically, since GSH is an essential cofactor for GPX4, its depletion inactivates this key ferroptosis suppressor. Western blot analysis confirmed significant downregulation of GPX4 protein in tumor cells treated with CMP/CMAP MOFs (Fig. 2B&C). This GSH depletion-mediated GPX4 inactivation disrupts cellular redox balance, impairing lipid peroxide repair. Consequently, the generation of ⋅OH in cancer cells was detected using the DCFH-DA probe. Both CMP MOFs and CMAP MOFs groups exhibited stronger green fluorescence compared to the BLANK group (Fig. 2D-E&S6). Production of ⋅OH intensified in a dose-dependent manner, as evidenced by progressively brighter fluorescence with increasing CMP MOFs or CMAP MOFs concentrations (Fig. S7). This oxidative stress contributed to lipid peroxidation, a hallmark of ferroptosis, as assessed by BODIPY 581/591 C11. In CMP MOFs and CMAP MOFs groups, oxidized lipids displayed brighter green fluorescence, while reduced lipids exhibited darker red fluorescence compared to the BLANK group (Fig. 2F). Flow cytometry analysis further revealed a significantly higher Oxidized/Reduced lipid ratio in CMP MOFs and CMAP MOFs groups compared to the BLANK group, demonstrating efficient lipid peroxidation (Fig. 2G&H). Additionally, the expression of PTGS2, a ferroptosis marker, was substantially upregulated in CMP MOFs and CMAP MOFs groups (Fig. 2I&J), confirming effective ferroptosis induction through both ⋅OH generation and lipid peroxide repair defection.

### In vitro assessment of CMAP MOFs-mediated immunogenicity enhancement

**Fig. 3:**
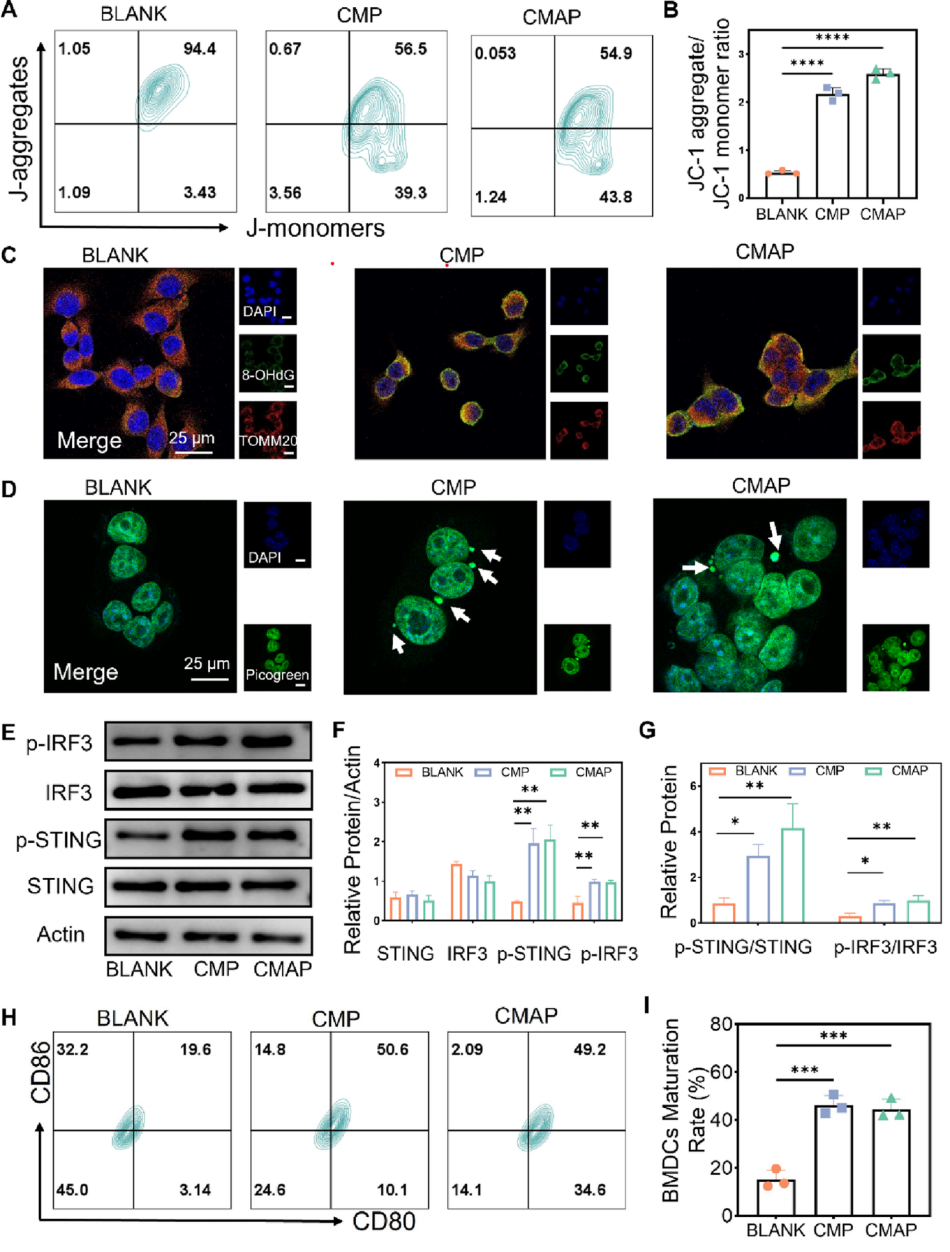
In vitro assessment of CMAP MOFs-mediated immunogenicity enhancement. (**A**) Corresponding flow cytometry analyses of JC-1 assay of 4T1 cells treated with different groups (**B**) and quantitative analysis. (**C**) Representative immunofluorescence images showing mtDNA oxidation levels inside treated 4T1 cells stained with TOMM20 (red), 8-OHdG (green), and DAPI (blue), scale bar: 25 μm. (**D**) Representative images of picogreen-stained 4T1 cells after treatment with different groups (the white arrow stands for escaped dsDNA). (**E**) Western blot analysis of cGAS-STING related proteins (including STING, IRF3, p-STING, and p-IRF3) from BMDCs and (**F**) relative quantification of STING, IRF3, p-STING, p-IRF3 in BMDCs. (**G**) The relative expression of p-STING/STING and p-IRF3/IRF3 proteins in BMDCs. (**H**) Flow cytometry analysis of BMDCs maturation after different treatments (**I**) and quantitative statistical analysis. *p < 0.05, **p < 0.01, ***p < 0.001, ****p < 0.0001

As the primary target organelle of ⋅OH/ferroptosis mediated damage, mitochondria are likely to sustain significant damage following treatment with CMP MOFs or CMAP MOFs. JC-1 staining coupled with flow cytometry analysis revealed a significant decrease in mitochondrial membrane potential in cancer cells treated with CMP MOFs or CMAP MOFs (Fig. 3A-B & S8), confirming substantial mitochondrial damage. Due to the lack of histone protection, mtDNA is highly susceptible to oxidative stress and becomes oxidized, forming ox-mtDNA. Concurrently, using 8-hydroxy-2’-deoxyguanosine (8-OHdG) and translocase of outer mitochondrial membrane 20 (TOMM20) as probes, a marked increase in ox-mtDNA within the treated cancer cells was observed (Fig. 3C). As a result, the PicoGreen assay revealed a significant increase in double-stranded DNA (dsDNA) within the cytoplasm of the CMP MOFs and CMAP MOFs groups (Fig. 3D). Collectively, these findings demonstrate the ability of CMP MOFs and CMAP MOFs to induce mitochondrial damage via oxidative stress, which in turn promotes the cytoplasmic release of dsDNA.

It is well-established that ox-mtDNA serves as a key molecule for activating the cGAS-STING pathway, and this activation could be significantly enhanced in the presence of Mn²⁺ by facilitating the production of cyclic GMP-AMP (cGAMP) and strengthening cGAMP/STING binding affinity [23, 34–36]. To validate the efficacy of CMP MOFs and CMAP MOFs in activating the cGAS-STING pathway, bone marrow-derived dendritic cells (BMDCs) were co-incubated with the supernatant obtained from 4T1 cells treated with CMP MOFs or CMAP MOFs. Western blot analysis revealed significantly upregulated expression of phosphorylated STING (p-STING) and phosphorylated interferon regulatory factor 3 (p-IRF3), the core components of the cGAS-STING signaling cascade, in BMDCs (Fig. 3E-G). The secretion level of IFN-β was also significantly increased in the CMP MOFs and CMAP MOFs (Fig. S9). Subsequently, flow cytometry analysis detected elevated surface expression of the maturation markers CD80 and CD86 on BMDCs (Fig. 3H-I). Collectively, these results demonstrate that CMAP MOFs effectively enhance tumor immunogenicity through mitochondrial damage-induced ox-mtDNA release and subsequent cGAS-STING pathway activation, ultimately promoting DC maturation and immune activation.

### In vivo antitumor efficacy and biosafety evaluation

Prior to assessing the antitumor efficacy in vivo, the biodistribution of CMAP MOFs was validated in BALB/c mice. Firstly, CMAP MOFs were labeled with IR780 to track their biodistribution. The fluorescence intensity at the tumor site increased progressively, peaking 24 h post-intravenous injection (Fig. S10), demonstrating favorable tumor accumulation of CMAP MOFs for precision therapy. To quantitatively analyze tissue distribution, we used ICP-MS to measure copper levels (the key metal component in CMAP MOFs) in major organs (heart, liver, spleen, lungs, kidneys, and tumors) at 1, 6, 12, 24, and 48 h post-injection, with three samples collected at each time point (*n* = 3). As shown in Fig. S11, CMAP MOFs were primarily enriched in liver and tumor tissues, consistent with the typical in vivo distribution characteristics of nanoparticles. Tumor accumulation gradually increased, peaking 24 h after injection, and then gradually declined, which aligns with our in vivo imaging results.

**Fig. 4:**
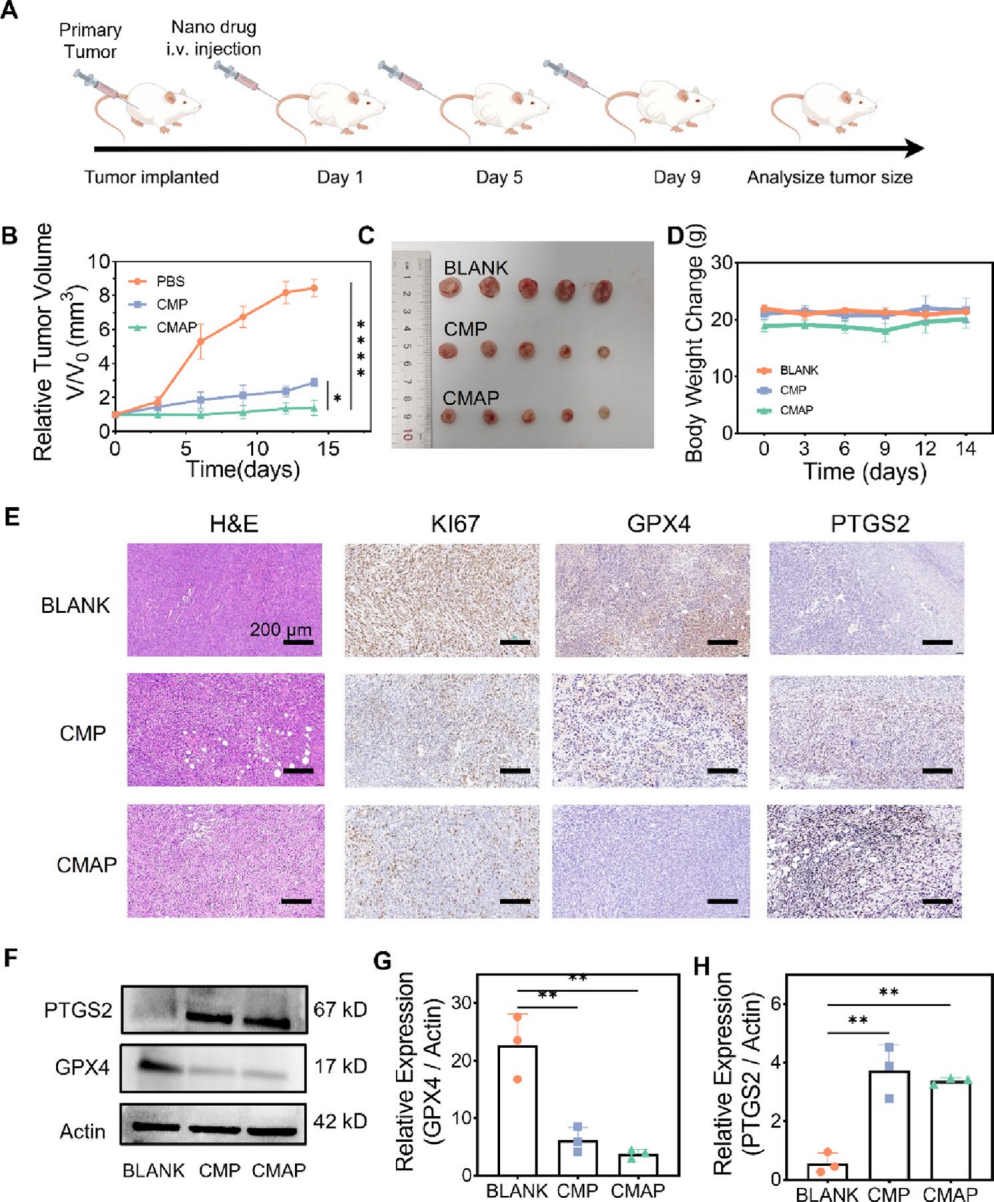
In vivo antitumor efficacy and biosafety evaluation in 4T1 tumor model. (**A**) Schematic illustration and timeline of the treatment protocol using the as-prepared MOFs. (**B**) Relative tumor growth curves. (**C**) Representative photographs of 4T1 xenograft tumors from mice after different treatments (*n* = 5). (**D**) Body weight changes of mice after the different treatments (*n* = 5). (**E**) Representative images of immunohistochemical staining (Ki67, GPX4 and PTGS2) and hematoxylin-eosin staining (H&E) of 4T1 xenograft tumors with the indicated treatments. Scale bars: 200 μm. (**F**) Western blot analysis of GPX4 and PTGS2 from treated tumors and (**G**) (**H**) quantitative analysis. Data are presented as mean ± SD (*n* = 3). *p < 0.05, **p < 0.01, ****p < 0.0001

To thoroughly assess the therapeutic efficacy of CMAP MOFs in vivo, 4T1 tumor bearing mice were randomized into three treatment groups receiving intravenous injections of PBS, CMP MOFs, or CMAP MOFs on days 1, 5, 9, and 13 (Fig. 4A). Tumor growth analysis revealed rapid progression in PBS controls, moderate suppression with CMP MOFs (74.1% inhibition rate), and potent inhibition with CMAP MOFs (81.3% inhibition rate) (Fig. 4B & C). No substantial variation in body weight was observed among all treatment groups of mice, indicating favorable biosafety of CMAP MOFs (Fig. 4D). H&E and Ki67 staining revealed the most pronounced tumor necrosis and minimal Ki67^+^ proliferating cells in the CMAP MOFs group, consistent with its superior antitumor activity (Fig. 4E). Furthermore, CMAP MOFs group exhibited significant downregulation of GPX4 and concurrent significant upregulation of PTGS2 (Fig. 4F-H), confirming effective ferroptosis induction in vivo.

Given the clinical translation potential of nanoagents, comprehensive biosafety validation is essential. Biosafety was evaluated using hemolytic assays and H&E staining of major organs (heart, liver, spleen, lung, and kidney). As shown in Fig. S12, CMAP MOFs exhibited negligible hemolysis (< 5%) even at the maximum tested concentration (1000 µg/mL). Additionally, H&E staining of major organs harvested 72 h post treatment revealed no significant histopathological changes in CMP/CMAP MOFs (Fig. S13). Kidney (creatinine, CR), heart (creatine kinase, CK) and liver (alanine aminotransferase, ALT) function biomarkers in healthy mice showed no significant variation across all groups (Fig. S14), collectively confirming the excellent biocompatibility of CMAP MOFs.

### Synergistic antitumor immunity through combined immunogenicity enhancement and immunosuppression relief

Inspired by the satisfactory antitumor efficacy, we proceeded to investigate the underlying mechanisms. Based on our hypothesis, it was hypothesized that the antitumor mechanism primarily involves two aspects, including cGAS-STING pathway activation and amelioration of PMN-MDSCs ferroptosis. Therefore, mechanistic validation studies were performed to confirm this dual immunomodulatory strategy.

**Fig. 5:**
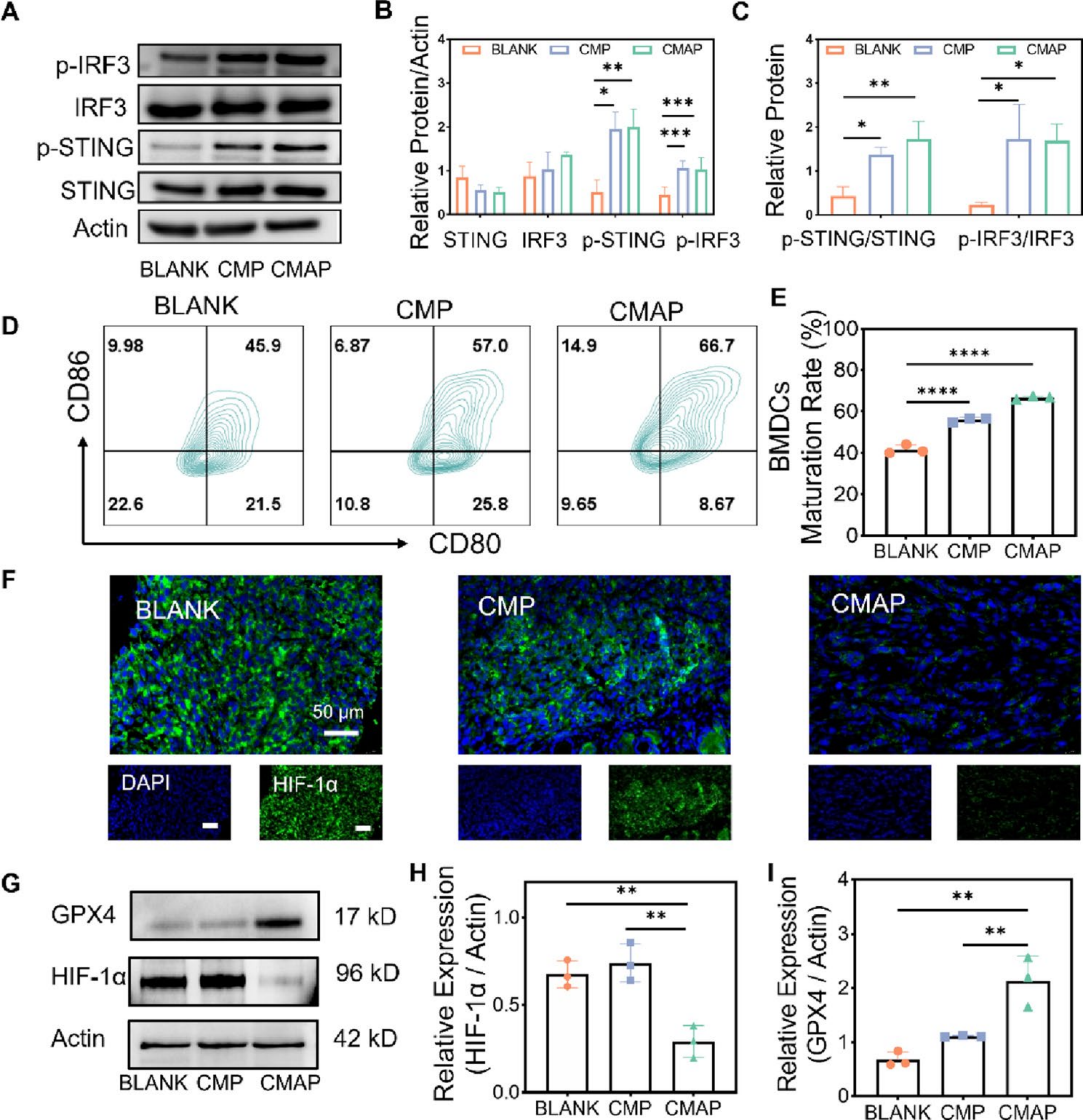
CMAP MOFs-mediated cGAS-STING pathway activation and PMN-MDSCs ferroptosis mitigation. (**A**) Western blot analysis of cGAS-STING related proteins (including STING, IRF3, p-STING and p-IRF3) from treated tumors and (**B**) relative quantification of STING, IRF3, p-STING, p-IRF3 from treated tumors. (**C**) The relative expression of p-STING/STING and p-IRF3/IRF3 proteins from treated tumors. (**D**) Representative flow cytometry analysis of BMDCs (CD45 + CD11c+CD80 + CD86+) in tumor samples extracted from mice after different treatments and (**E**) statistical analysis. (**F**) HIF-1α staining of tumors post-treatment. Scale bar: 50 μm. (**G**) Western blot analysis of HIF-1α and GPX4 in PMN-MDSCs derived from tumor tissues (**H**) (**I**) and quantitative analysis. *p < 0.05, **p < 0.01, ***p < 0.001, ****p < 0.0001

Western blot results showed elevated p-STING and p-IRF3 expression in the CMP/CMAP MOFs treated groups (Fig. 5A-C), with parallel ELISA and immunohistochemical staining confirmed robust IFN-β secretion (Fig. S15), indicating effective cGAS/STING pathway activation. Correspondingly, DC maturation was significantly enhanced in both CMP MOFs (57.0%) and CMAP MOFs (66.7%) groups compared to the Blank group (45.9%), with CMAP MOFs showing further improvement over CMP MOFs (Fig. 5D & E).

In addition to cGAS-STING pathway activation, immunosuppression represents another critical factor affecting antitumor immune efficacy. It has been reported that PMN-MDSCs within the hypoxic TME exhibit higher susceptibility to ferroptosis [30]. Hypoxia TEM downregulates GPX4 expression in PMN-MDSCs, compromising their resistance to ferroptosis. The lipid peroxides leaked from ferroptotic PMN-MDSCs contribute to T-cell inactivation and immunosuppression in cancer [30, 37]. To validate this hypothesis, PMN-MDSCs were first isolated from mouse tumor tissues with a purity of approximately 96%. (Fig. S16). CD71, a recognized ferroptosis marker, was used to assess neutrophil ferroptosis levels via flow cytometry [38]. The flow cytometry results showed that a significant increase in CD71 expression in intratumoral PMN-MDSCs compared to splenic neutrophils (Fig. S17), demonstrating that intratumoral PMN-MDSCs were more susceptible to ferroptosis. Fig. S18–S19 shows the gating strategy of flow cytometry for detecting CD71 expression on PMN-MDSCs in spleens and tumors of 4T1 tumor-bearing mice.

Subsequently, the efficacy of CMAP MOFs in ameliorating tumor hypoxia and thus reducing PMN-MDSCs ferroptosis within the TME was evaluated. Immunofluorescence analysis revealed notably reduced green fluorescence intensity of HIF-1α in tumor area from the CMAP MOFs group(Fig. 5F). Correspondingly, Western blot analysis displayed significantly diminished expression of the hypoxia-responsive protein (HIF-1α) in intratumoral PMN-MDSCs from CMAP MOFs treated mice compared to CMP MOFs and BLANK groups (Fig. 5G and H), confirming that CMAP MOFs could alleviate the hypoxic microenvironment in vivo. Moreover, PMN-MDSCs isolated from CMAP MOFs-treated mice exhibited higher levels of GPX4 relative to BLANK and CMP MOFs groups (Fig. 5G and I), demonstrating that CMAP MOFs diminish ferroptosis susceptibility in PMN-MDSCs through hypoxia alleviation.

**Fig. 6:**
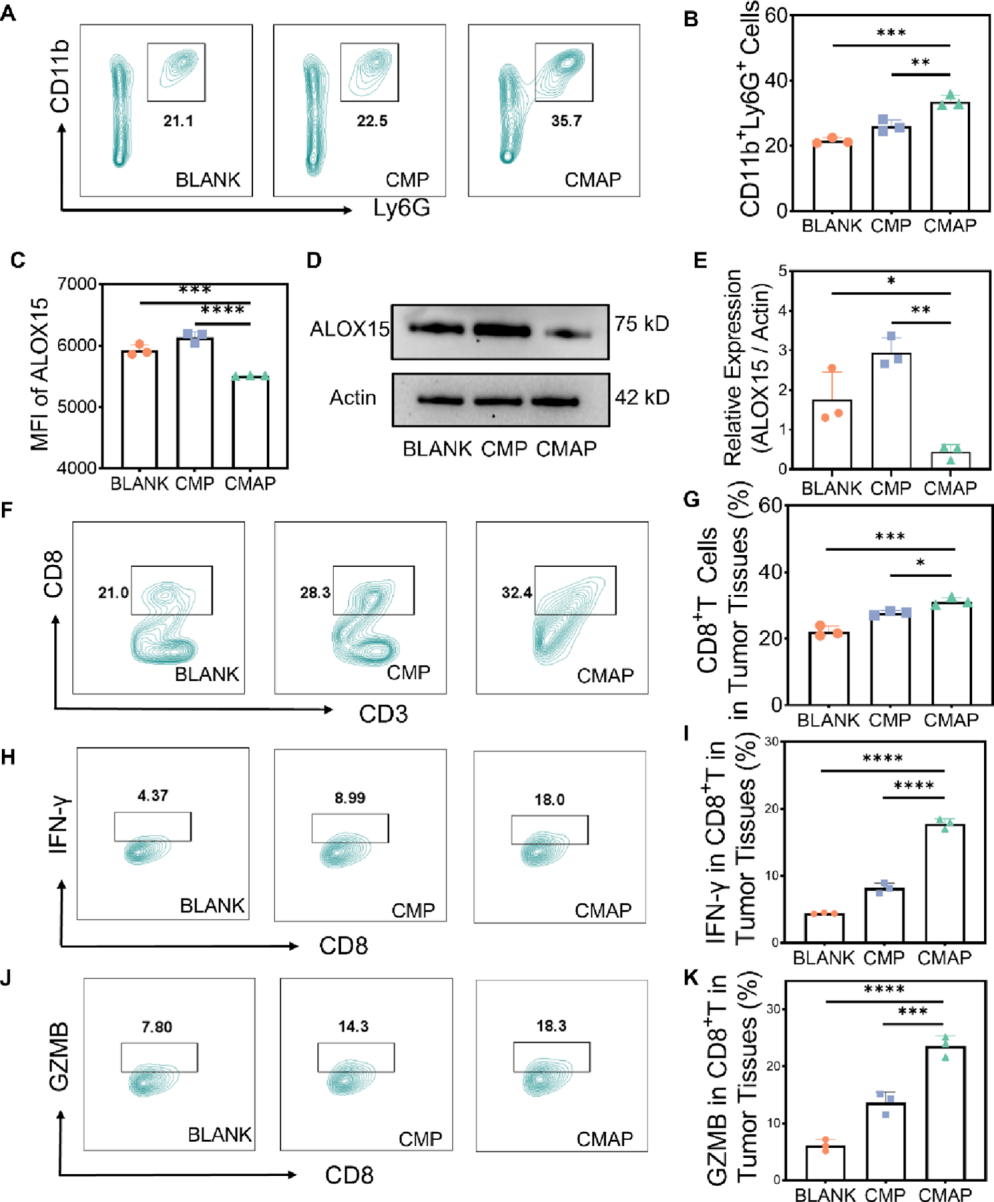
Systemic Immune Activation by CMAP MOFs. (**A**) Flow cytometry analysis of PMN-MDSCs (CD45+CD11b+Ly6G+) in tumor samples extracted from mice after different treatments and (**B**) statistical analysis of PMN-MDSCs. (**C**) ALOX15 expression of PMN-MDSCs in tumor tissues determined by flow cytometry after different treatments. (**D**) Western blot analysis of ALOX15 in PMN-MDSCs derived from tumor tissues and (**E**) quantitative analysis. (**F**) Flow cytometry analysis of CD8+ T cells (CD45+CD3+CD8+) in tumor samples extracted from mice after different treatmentsand and (**G**) statistical analysis. (**H**) Flow cytometry analysis of IFN-γ production in CD8 + T cells (CD45+CD3+CD8+) from tumor samples after different treatments and (**I**) statistical analysis. (**J**) Flow cytometry analysis of GZMB production in CD8+ T cells (CD45+CD3+CD8+) from tumor samples after different treatments and (**K**) statistical analysis. *p < 0.05, **p < 0.01, ***p < 0.001, ****p < 0.0001

Since reduced ferroptosis in PMN-MDSCs mediated by CMAP MOFs triggered hypoxia alleviation in the TME, the downstream immunological consequences were further investigated. Flow cytometry and immunohistochemical staining revealed substantially increased PMN-MDSCs infiltration in CMAP MOFs-treated tumors compared to BLANK and CMP MOFs groups (Fig. 6A and B & S20). To further elucidate the mechanism underlying PMN-MDSCs functional modulation, arachidonate-15-lipoxygenase (ALOX15) was examined, as it acts as a central executor of ferroptosis by directly catalyzing lipid peroxidation and endows PMN-MDSCs with immunosuppressive function [39]. Flow cytometric and western blot analyses confirmed that CMAP MOFs treatment significantly downregulated ALOX15 expression in PMN-MDSCs (Fig. 6C-E), demonstrating that CMAP MOFs modulated the ALOX15 pathway to influence ferroptosis and afterwards immunosuppression.

To further verify that the reversal of immunosuppression is associated with ALOX15, we conducted further experiments using the selective ALOX15 inhibitor ML351 in a 4T1 tumor-bearing mouse model. As shown in Fig. S21, both the CMP+ML351 and CMAP MOFs significantly downregulated CD71 expression in tumor-infiltrating PMN-MDSCs compared to the CMP MOFs, illustrating ALOX15 inhibition (CMP+ML351) and Au-mediated alleviation of hypoxic microenvironment (CMAP MOFs) both attenuated PMN-MDSCs ferroptosis. Consequently, the number of CD8 + T cells and level of the cytokine IFN-γ within the tumor significantly increased both in CMP+ML351 and CMAP MOFs, indicating the reversal of PMN-MDSCs ferroptosis-mediated immunosuppression (Fig. S22-S23). Collectively, these results suggested that PMN-MDSC ferroptosis generated immunosuppression through ALOX15, whereas CMAP MOFs reduced PMN-MDSCs ferroptosis sensitivity via Au-mediated alleviation of hypoxic microenvironment, ultimately inhibiting ALOX15 and reversing immunosuppression.

With both cGAS-STING pathway activation and PMN-MDSCs ferroptosis amelioration established, enhanced CD8⁺ T cell infiltration was identified (Fig. 6F and G & S24). The proportion of CD8^+^ T cells reached 28.3% in CMP MOFs group, representing a significant increase over the BLANK group and confirming cGAS/STING pathway-mediated antitumor immunity activation. Notably, the CMAP MOFs group exhibited further elevated CD8⁺ T cell infiltration levels (32.4%), demonstrating synergistic recruitment through relief of PMN-MDSCs immunosuppression and enhanced cGAS-STING activation.

Beyond infiltration, the functional capacity of CD8⁺ T cells was also assessed, as the release of interferon-γ (IFN-γ) and granzyme B (GZMB) by CD8⁺ T cells is crucial for effective immunotherapy. Flow cytometry analysis of IFN-γ and GZMB levels in tumor tissues revealed that CMAP MOFs treatment led to enhanced secretion of both cytokines compared to other groups (Fig. 6H-K). This immunomodulation elicited robust secretion of inflammatory cytokines (IL-6, TNF-α, IFN-γ) in CMAP MOFs-treated tumors compared to blank and CMP MOFs groups (Fig. S25-26).

Collectively, these findings demonstrate that CMAP MOFs promote antitumor immunity through synergistic enhancement of tumor immunogenicity *via* cGAS-STING activation and mitigating ferroptotic PMN-MDSCs induced immunosuppression.

## Conclusion

In summary, an innovative multifunctional nanoplatform (CMAP MOFs) was engineered comprising Cu/Mn bimetallic MOFs decorated with Au nanozymes for TNBC immunotherapy. CMAP MOFs disintegrate upon cleavage of disulfide bonds, selectively releasing Cu/Mn ions and catalytically active Au nanozymes. The released Cu/Mn ions catalyze Fenton-like reaction to generate toxic ROS, triggering tumor cell ferroptosis and ox-mtDNA release. Concurrently, Mn²⁺ ions serve as powerful amplifiers, significantly enhancing cGAS-STING pathway activation, promoting DC maturation, and increasing CD8⁺ T cell infiltration. On the other hand, the liberated Au nanozymes efficiently alleviate tumor hypoxia, thereby protecting PMN-MDSCs from ferroptosis and mitigating their immunosuppressive effects on T cell functionality. By synergistically activating the cGAS-STING pathway to enhance tumor immunogenicity and alleviate the immunosuppression induced by ferroptosis in polymorphonuclear leukocytes-myeloid-derived suppressor cells (PMN-MDSCs), the bifunctional CMAP metal-organic framework (MOF) nanoplatform we developed has demonstrated significant therapeutic efficacy. This strategy effectively converts immunologically “cold” tumors into “hot” tumors, characterized by a significant increase in CD8 + T-cell infiltration, enhanced cytotoxicity, and elevated levels of IFN-γ and GZMB secretion. These findings suggest that CMAP may possess the ability to induce long-term immune memory, future studies are needed to experimentally validate this capability, particularly through preclinical models assessing memory T-cell survival and tumor re-challenge experiments. In summary, combining enhanced immunogenicity with the alleviation of immunosuppression holds promise as an effective strategy to overcome immunotherapy resistance in patients with triple-negative breast cancer (TNBC) who have a poor prognosis.

## Supplementary Information

Below is the link to the electronic supplementary material.

Supplementary Material 1

## Data Availability

Data sharing is not applicable to this article as no datasets were generated or analysed during the current study. Data will be made available on request.
