# Supplementary material for: Gold nanozyme-decorated Cu/Mn MOFs simultaneously enhances immunogenicity and alleviates immunosuppression in triple-negative breast cancer
Source: J Nanobiotechnology. 2026 Apr 25;24:565. doi: 10.1186/s12951-026-04482-3 (PMC13274088; doi:10.1186/s12951-026-04482-3)
Supplement: Supplementary file 1 — Supplementary Material 1 [file 12951_2026_4482_MOESM1_ESM.docx]

**Supporting Information**

**Gold Nanozyme-Decorated Cu/Mn MOFs Simultaneously Enhances Immunogenicity and Alleviates Immunosuppression in Triple-Negative Breast Cancer**

Haibo Lan ^#^, Zede Wu^#^, Jinghua Xia ^#^, Qiuyu Li, Mengdan Gao, Zhuoxiu Cai, Weijing Tan, Minyi Liu, Ziting Xu, Yang Gao, Li Zhang, Bingxia Zhao*, Yingjia Li* and Yu Liang*

**
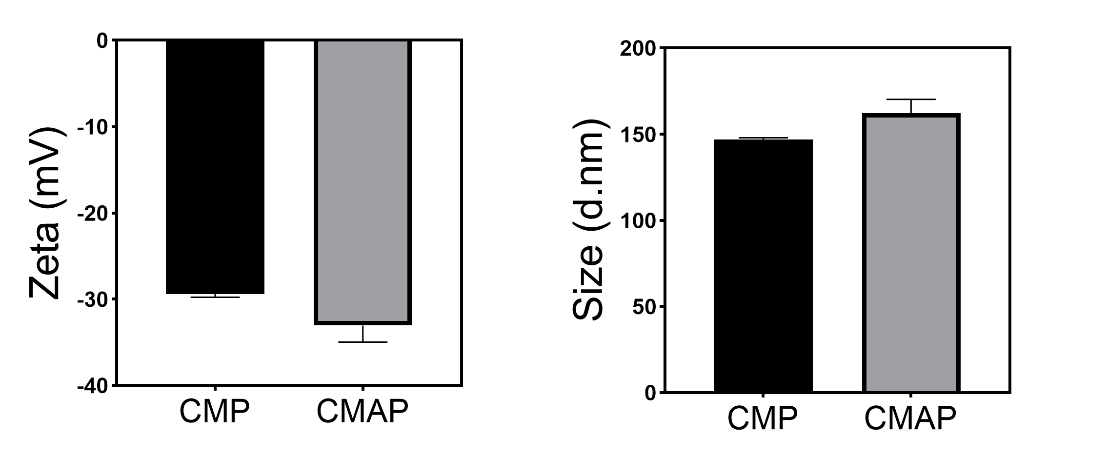
**

**Figure S1**. The Zeta potential changes and size of CMP MOFs and CMAP MOFs.


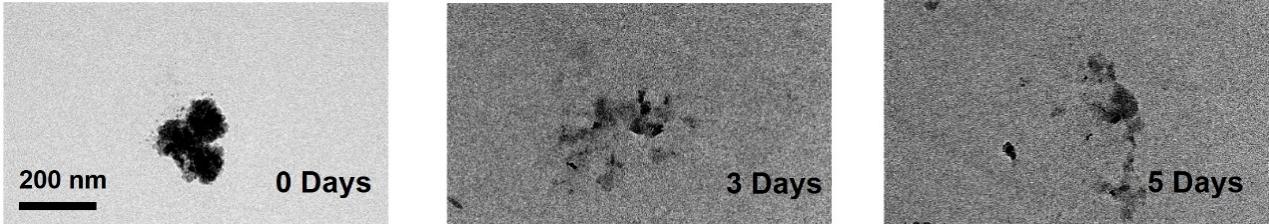


**Figure S2**. TEM images of biodegradable CMAP MOFs immersed in 10 mM GSH aqueous solution for 0 days, 3 days, and 5 days. Scale bar: 200 nm.


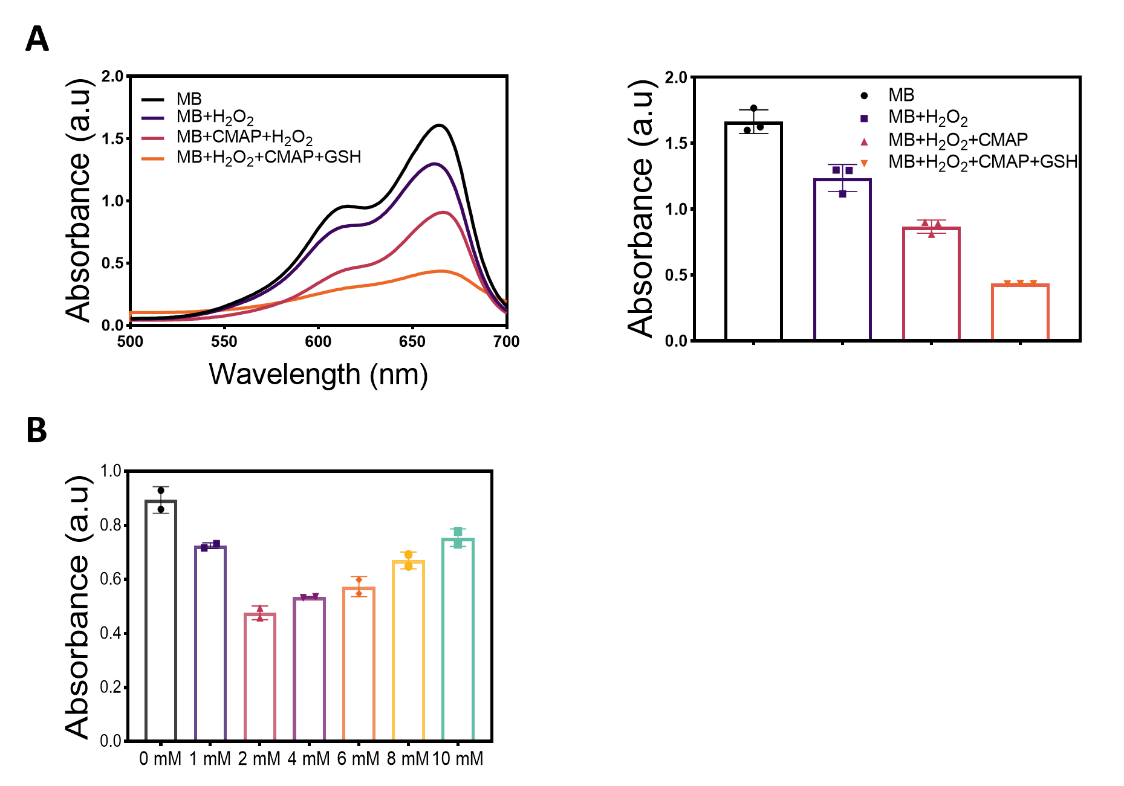


**Figure S3.** (A) Quantification of MB at 665 nm in CMAP MOFs solutions containing GSH (2 mM) and H_2_O_2_ (10 mM) and quantitative statistical analysis. Data were presented as mean ± SD (n = 3 for each group). (B) Quantification of MB at 665 nm in CMAP MOFs solutions with the existence of H_2_O_2_ (10 mM) and different concentration of GSH. Data were presented as mean ± SD (n = 3 for each group).


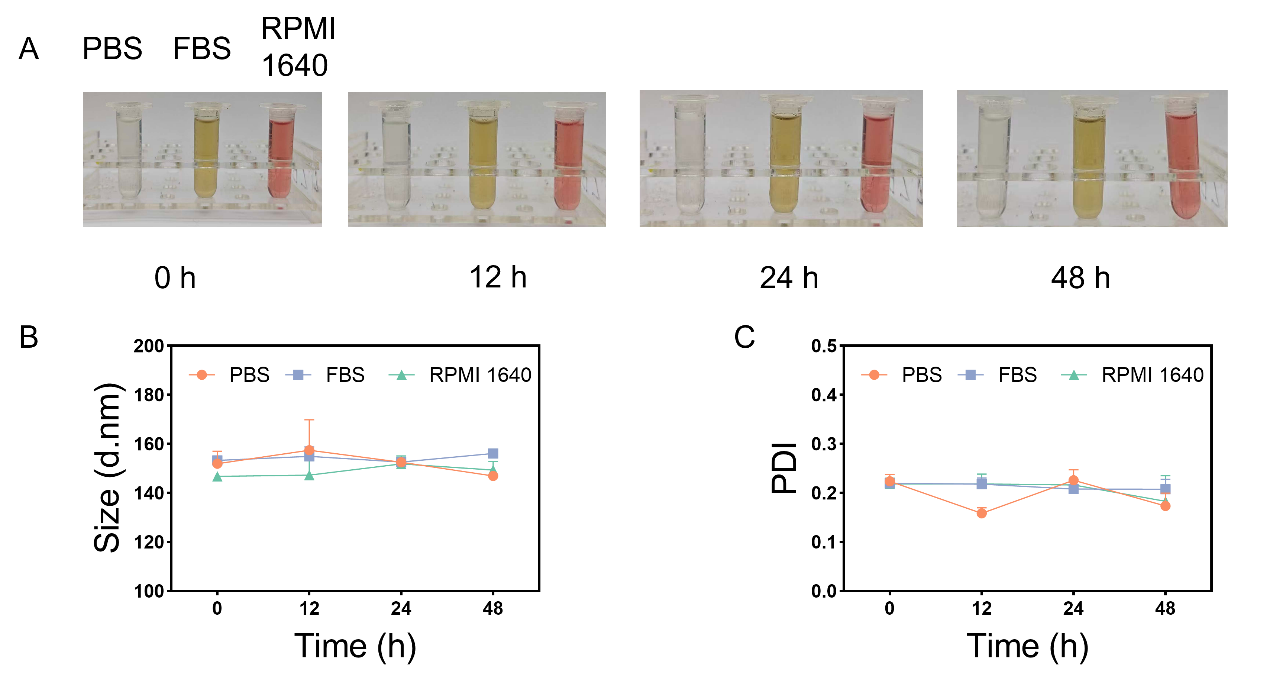


**Figure S4.** (A) Digital photographs of CMAP in different physiological solutions (From left to right: PBS, FBS and RPMI-1640). (B) The DLS results of various solutions containing CMAP. (C) The PDI results of various solutions containing CMAP. Data are presented as mean ± SD (*n* = 3).


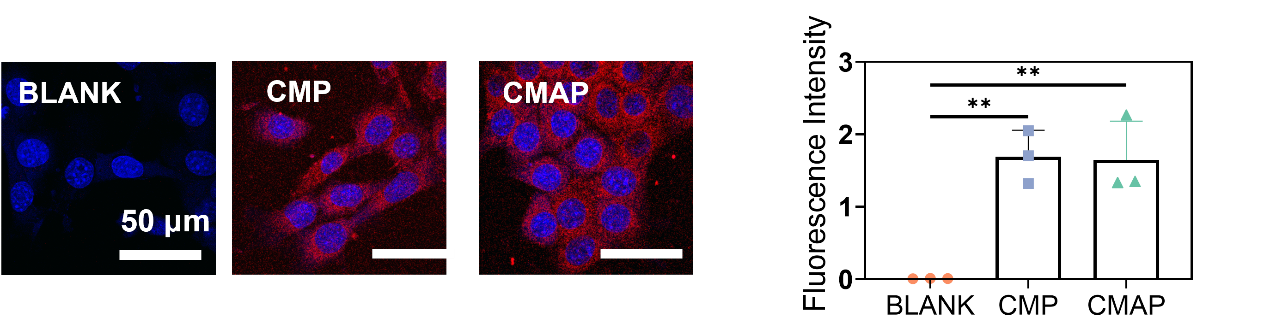


**Figure S5.** Representative CLSM images of 4T1 cells after incubation with RhB-labeled CMP MOFs and CMAP MOFs and quantitative statistical analysis. scale bar: 50 μm. **p < 0.01


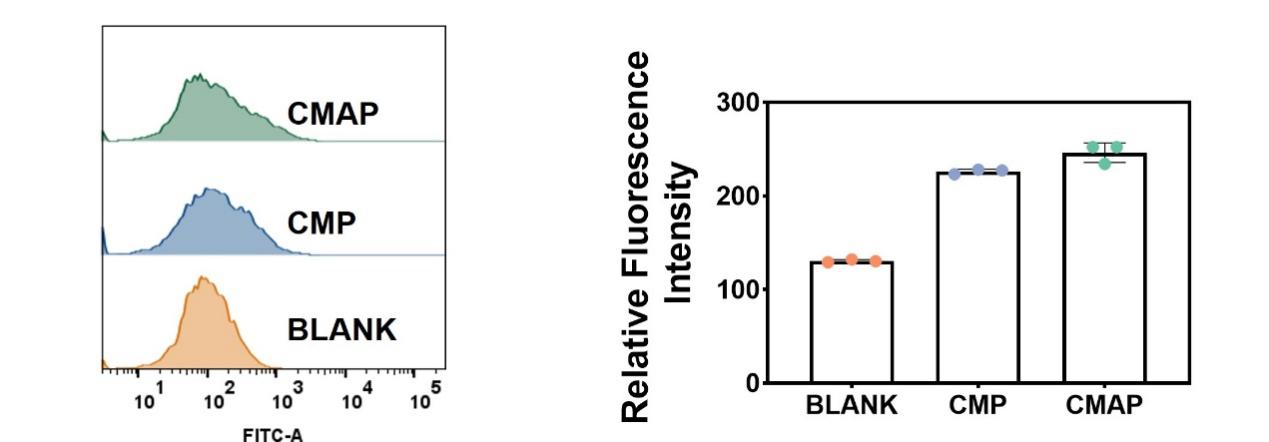


**Figure S6.** Corresponding flow cytometry analyses of DCFH-DA assay of 4T1 cells treated with different groups. Date are presented as mean ± SD (n ＝ 3).


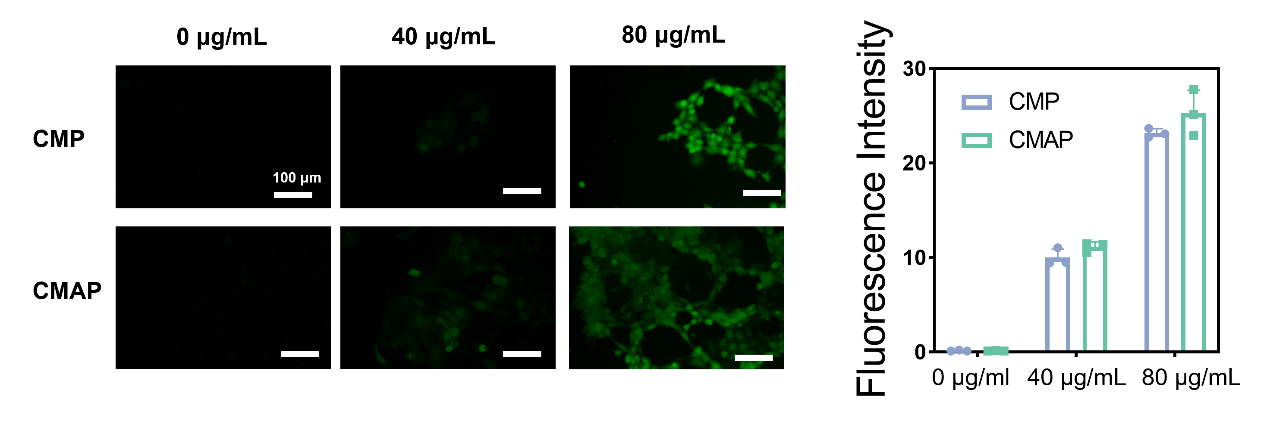


**Figure S7.** DCFH-DA assay of 4T1 cells treated with different concentration CMP MOFs and CMAP MOFs, scale bar: 50 μm.


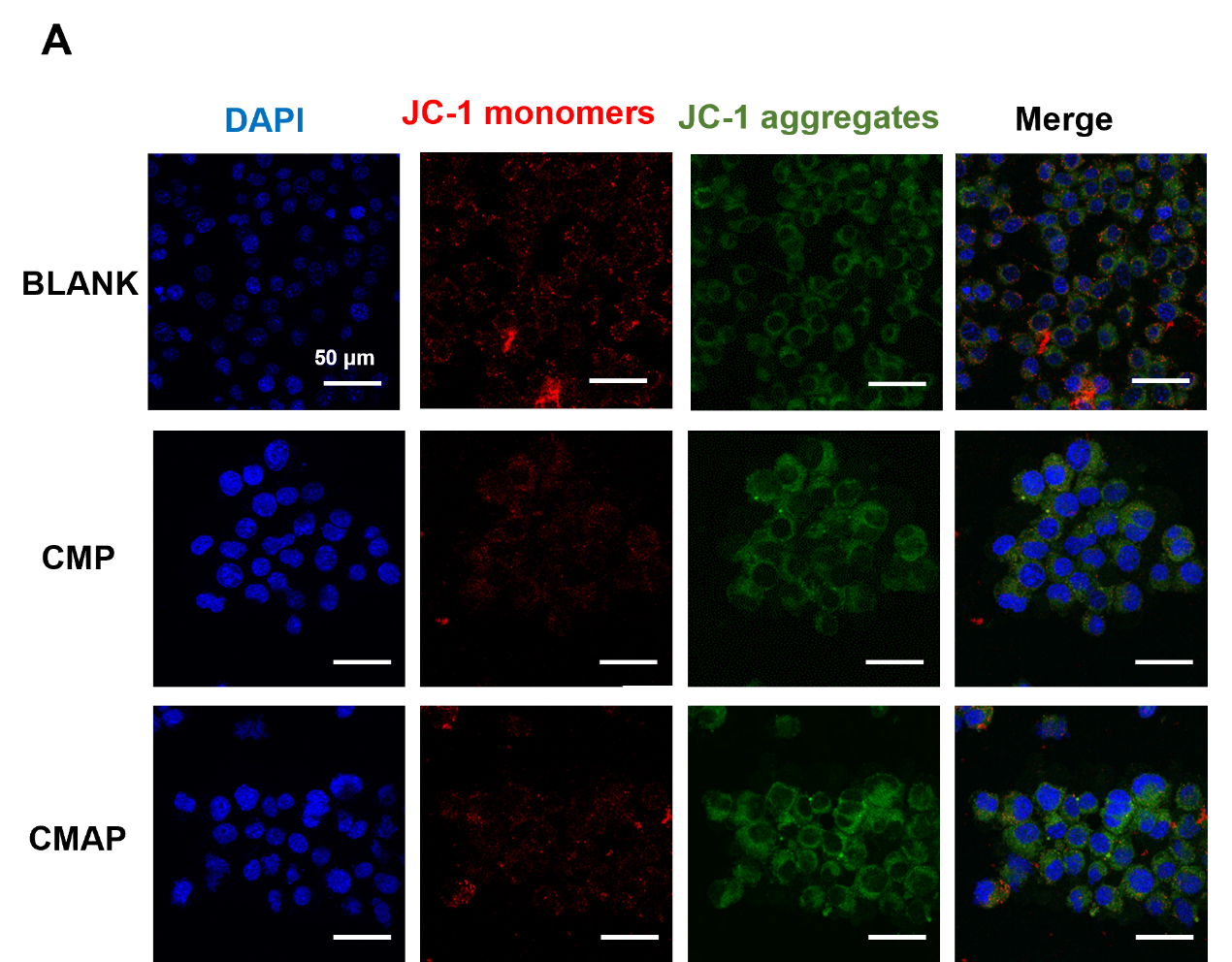


**Figure S8.** Representative immunofluorescence images of JC-1 of 4T1 cells after different treatments. Scale bar: 50 μm


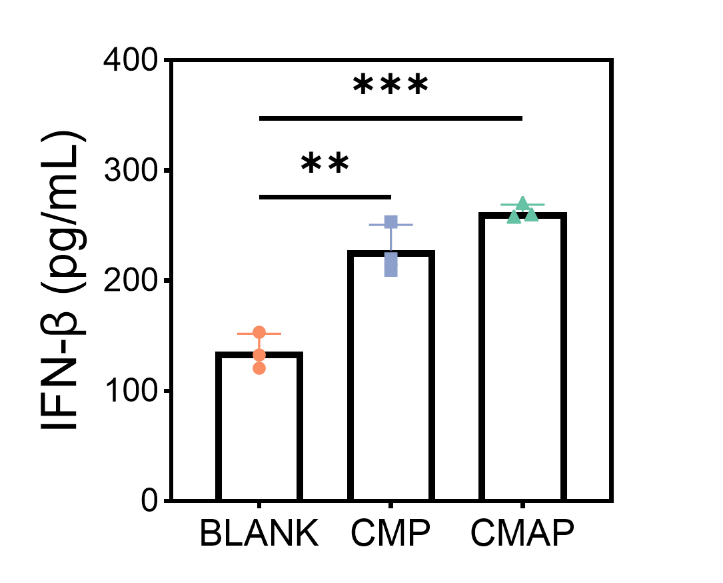


**Figure S9.** Cytokine levels IFN-β of DC cells under different treatments. Data are presented as mean ± SD (n = 3). **p < 0.01, ***p < 0.001.


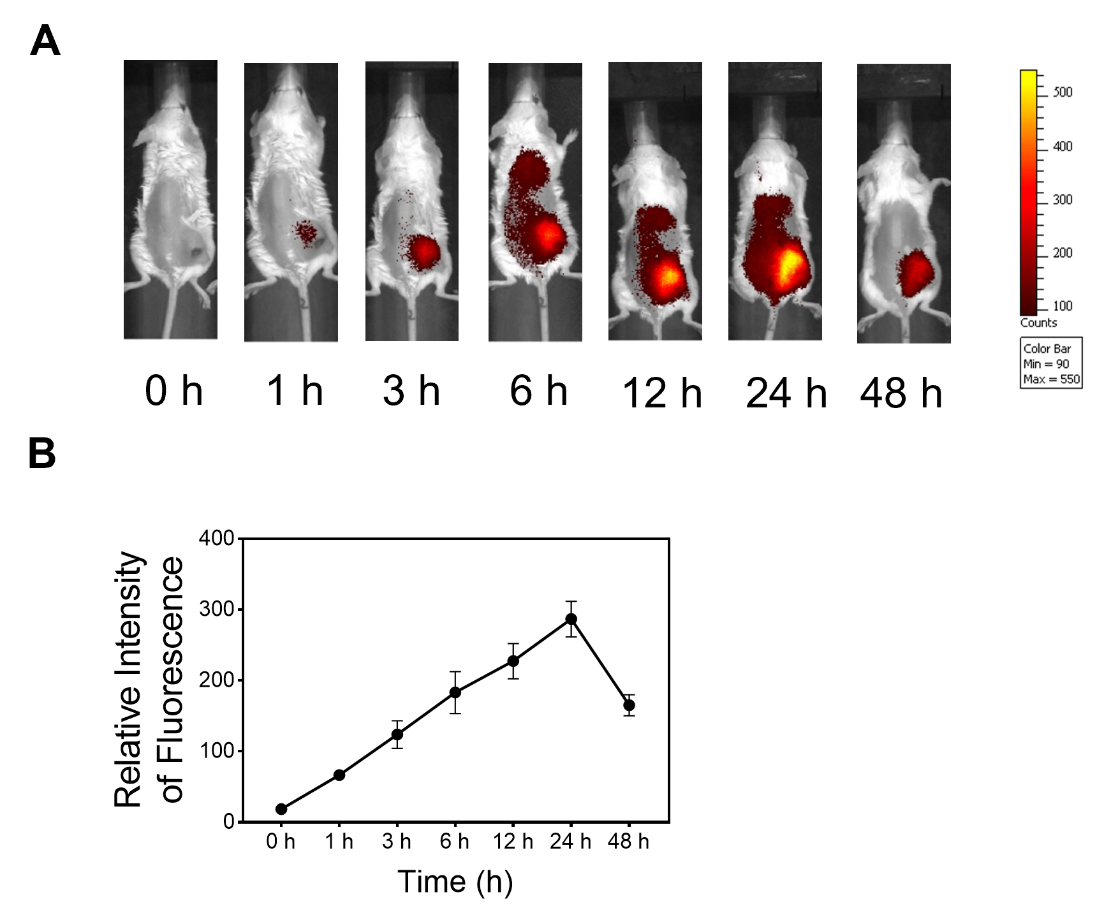


**Figure S10.** (A) In vivo fluorescence images of 4T1 tumor bearing mice at 0, 1, 3, 6, 12, 24,48 h after treatment with IR780 labeled CMAP MOFs (n = 3). and (B) quantitative analysis.

**
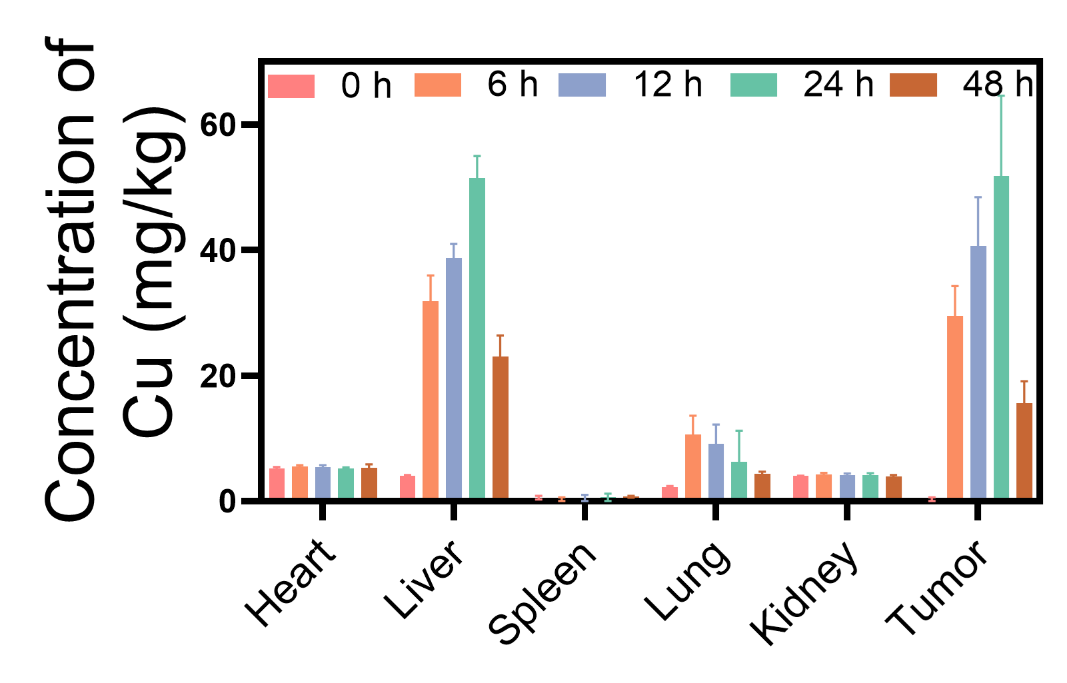
**

**Figure S11**. In vivo biodistribution of Cu levels from CMAP MOFs in 4T1 tumor-bearing mice.


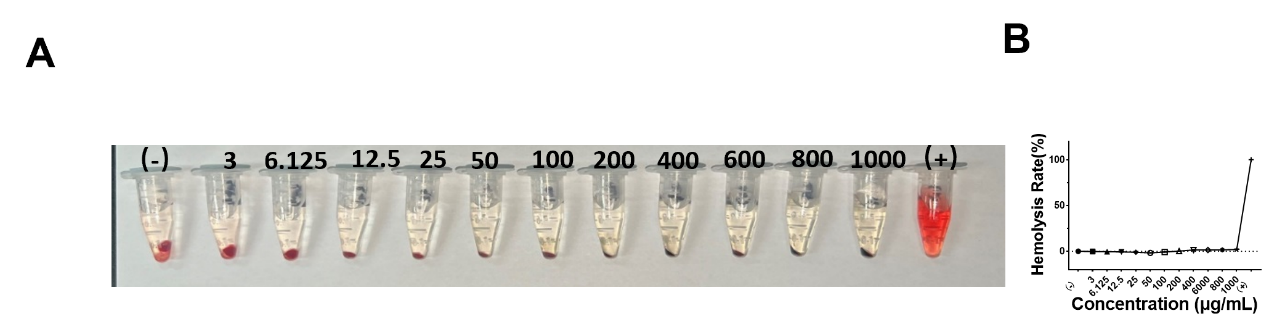


**Figure S12.** (A) Hemolysis experiment of CMAP MOFs after incubation with erythrocytes in various concentrations, deionized water (+) and PBS (-) were used as positive and negative controls, respectively. (B) Quantification of hemolysis for various concentrations.


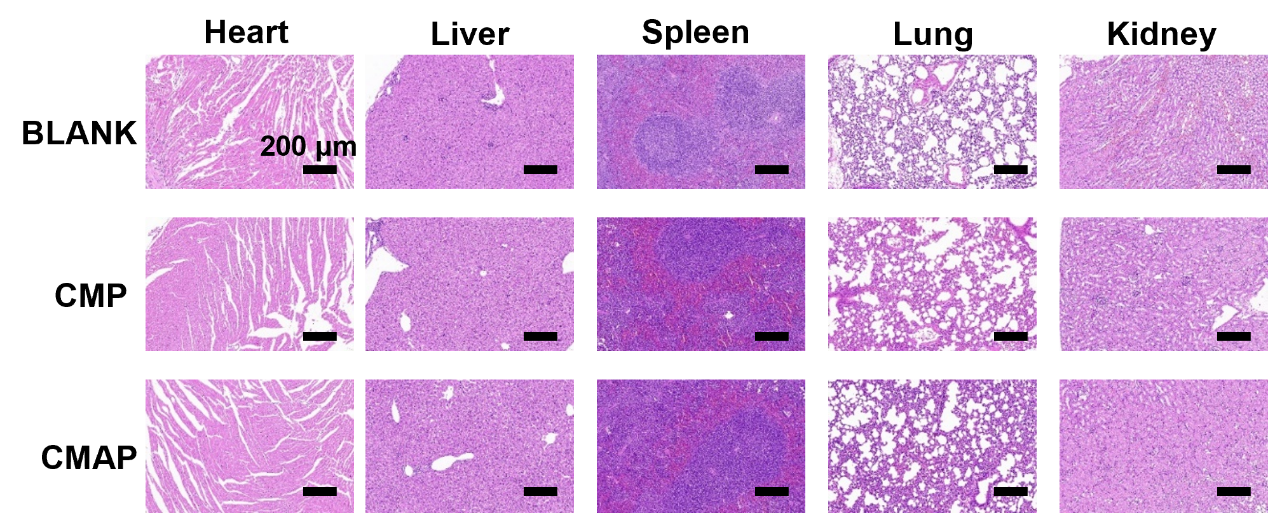


**Figure S13.** Representative image of H&E stained tissue section of the major organs. Scale bar: 200 µm.


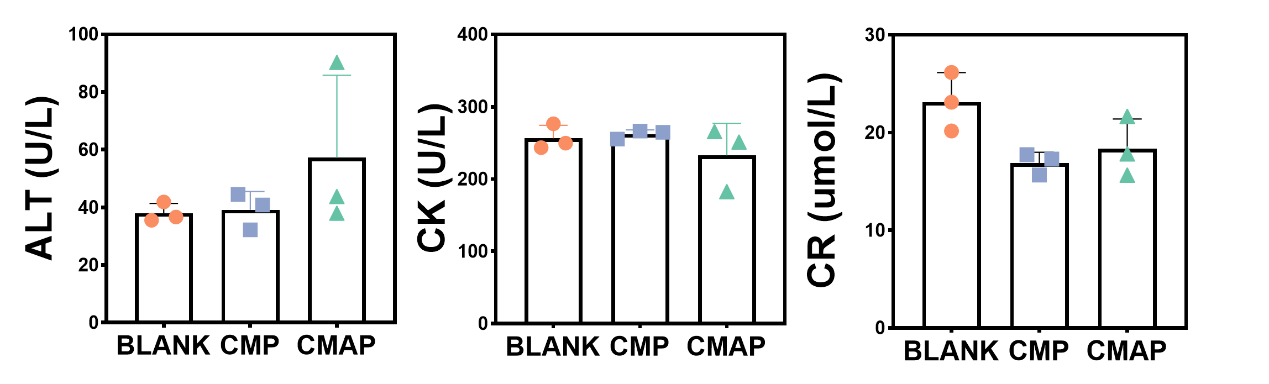


**Figure S14.** Biochemical assay results of serum collected from mice treated with PBS, CMP MOFs or CMAP MOFs. CR, creatinine; CK, creatine kinase; ALT, alanine aminotransferase.


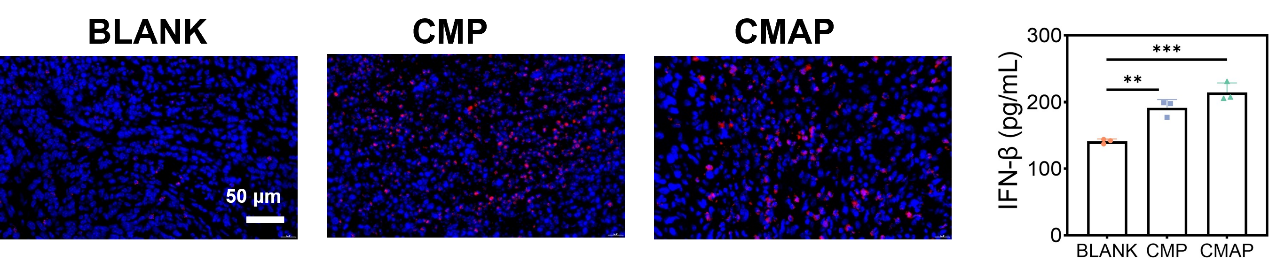


**Figure S15.** Immunofluorescence images of tumor sections for IFN-β(red) from mice treated with PBS, CMP MOFs or CMAP MOFs and cytokine levels of IFN-β in supernatants of tumors derived from treated mice. Data are presented as mean ± SD (n = 3). Scale bars: 50 μm. **p < 0.01, ***p < 0.001.


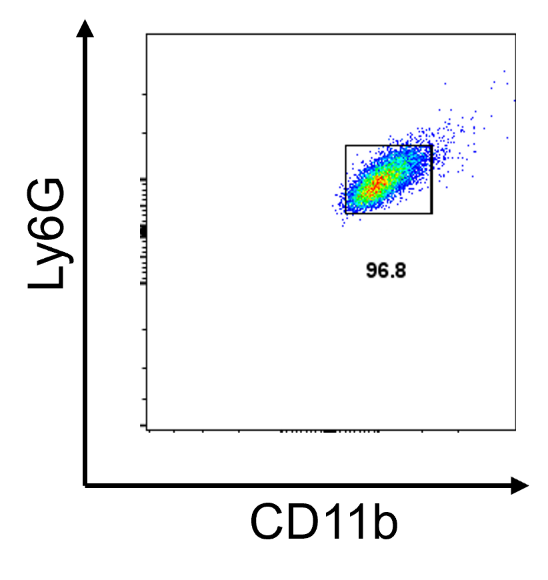


**Figure S16.** Purity of mouse PMN-MDSCs sorted from 4T1 tumors by flow cytometry.


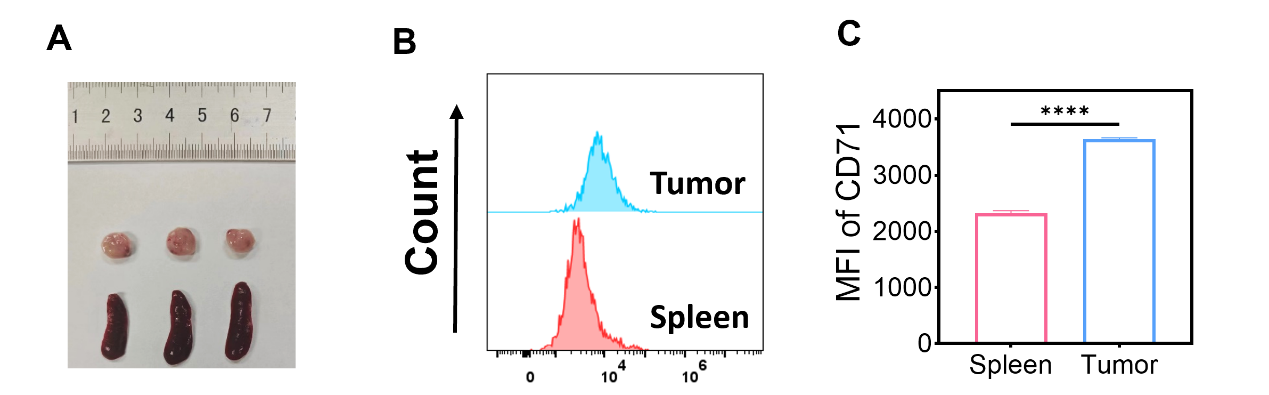


**Figure S17.** （A）Macroscopic image of tumor tissue and spleen from 4T1 tumor-bearing mice, n=3. (B) Ferroptosis of PMN-MDSCs detected by flow cytometry in TME and spleen of 4T1 tumor bearing mice without any treatments (C) and corresponding statistical analyses. ****p < 0.0001


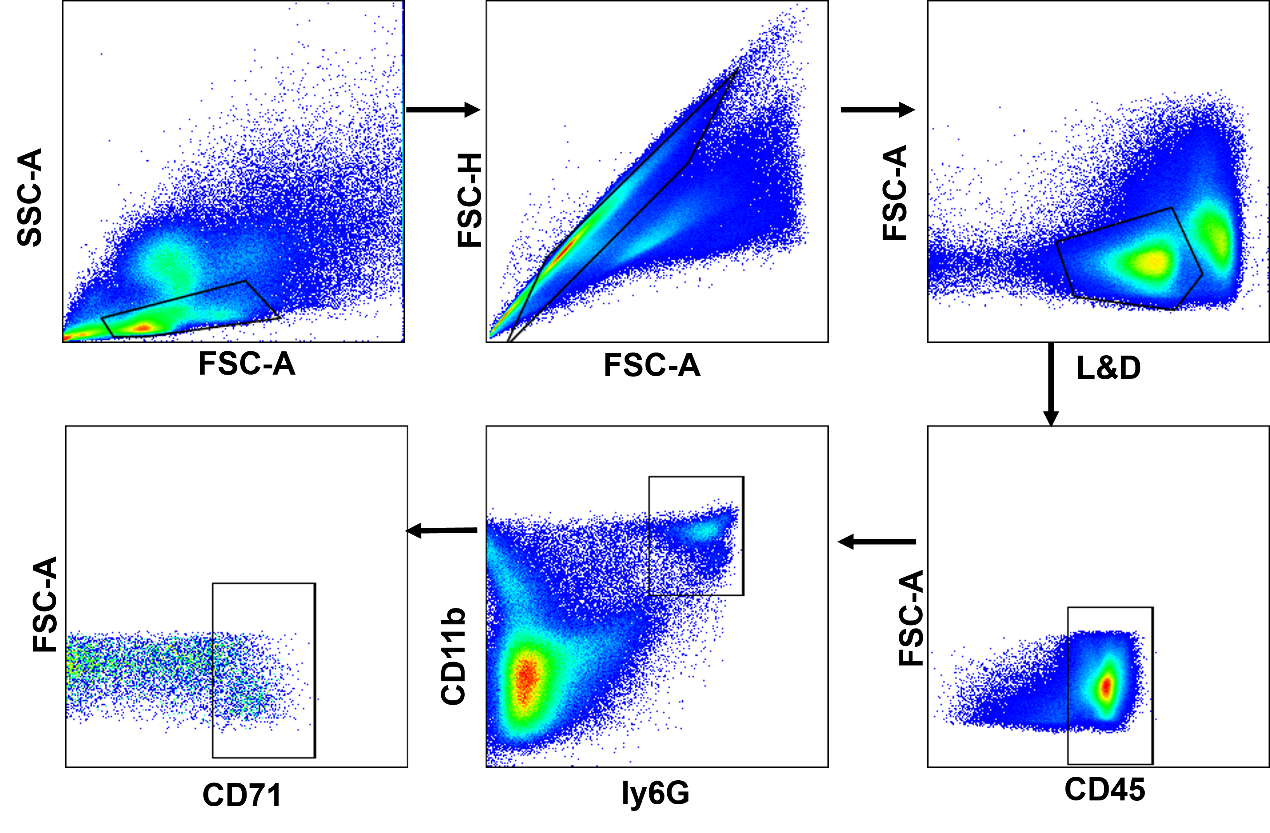


**Figure S18.** Gating strategies for CD71 expression on PMN-MDSCs in the spleen of 4T1 tumor-bearing mice via flow cytometry*.*


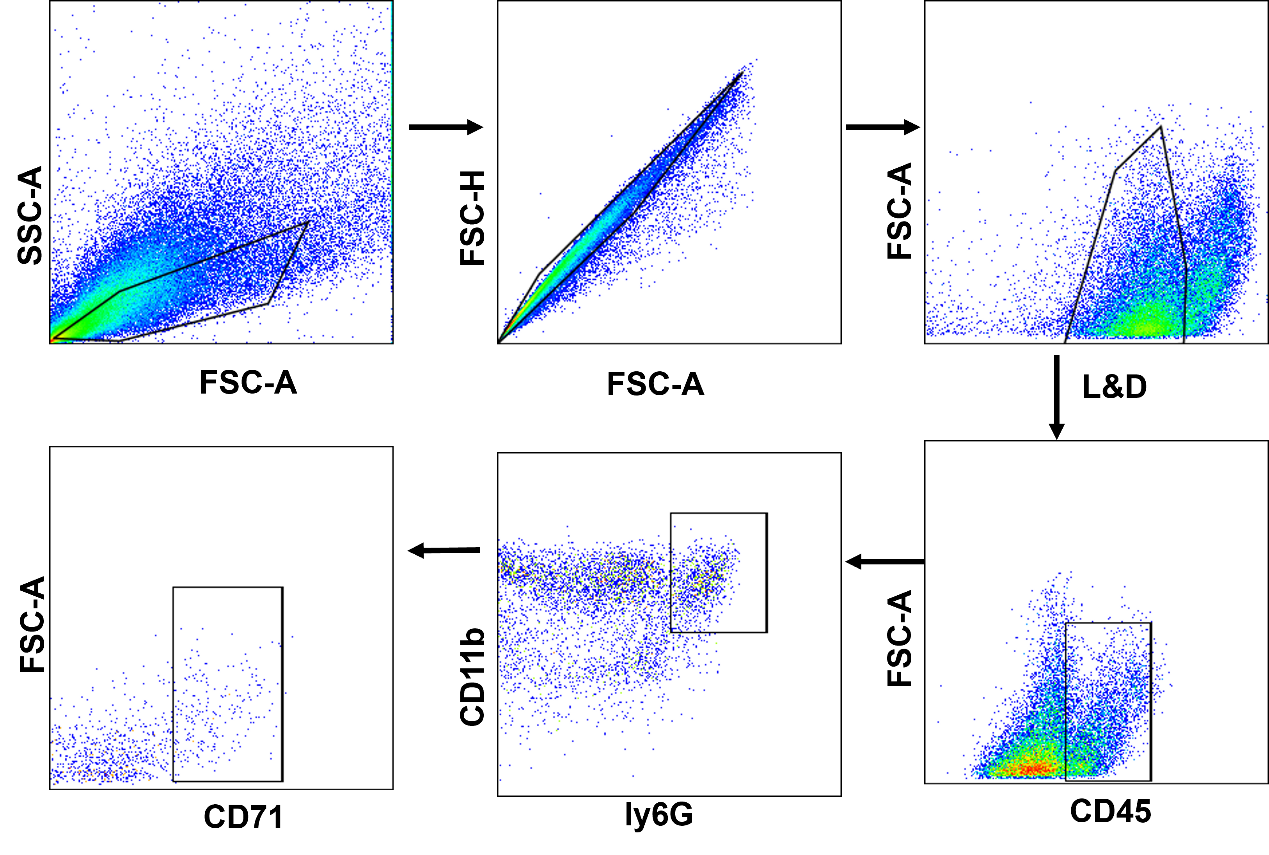


**Figure S19.** Gating strategies for CD71 expression on PMN-MDSCs in the tumor of 4T1 tumor-bearing mice via flow cytometry*.*


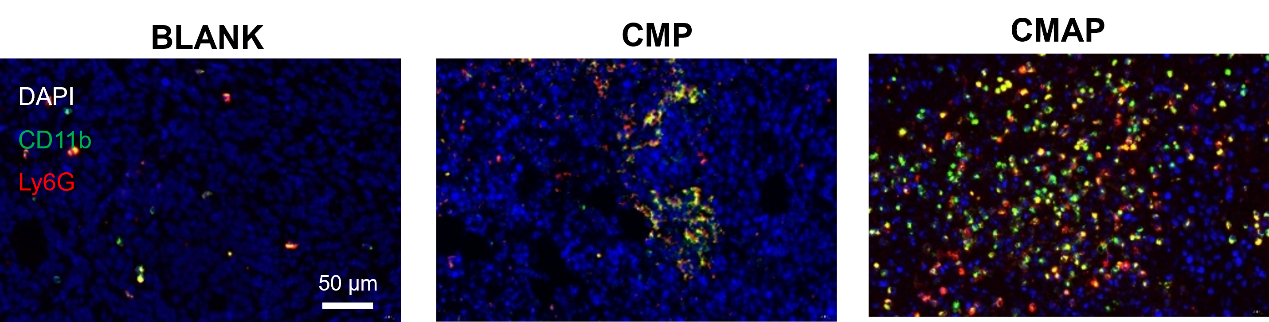


**Figure S20.** Immunofluorescence images of tumor sections of PMN-MDSCs stained with CD11b (green), or Ly6G (red), and DAPI (blue). Scale bars: 50 μm.


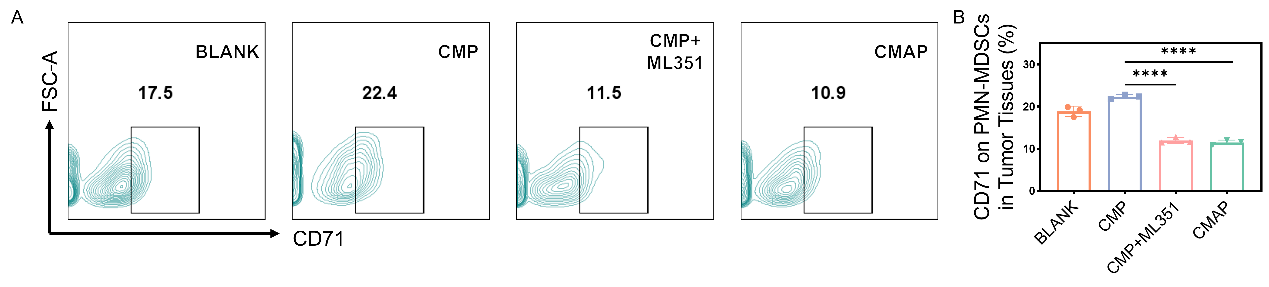


**Figure S21**. (A) Flow cytometry analysis of CD71 expression in PMN-MDSCs (CD45+CD11b+Ly6G+) from tumor samples after different treatments and (B) statistical analysis. ****p < 0.0001


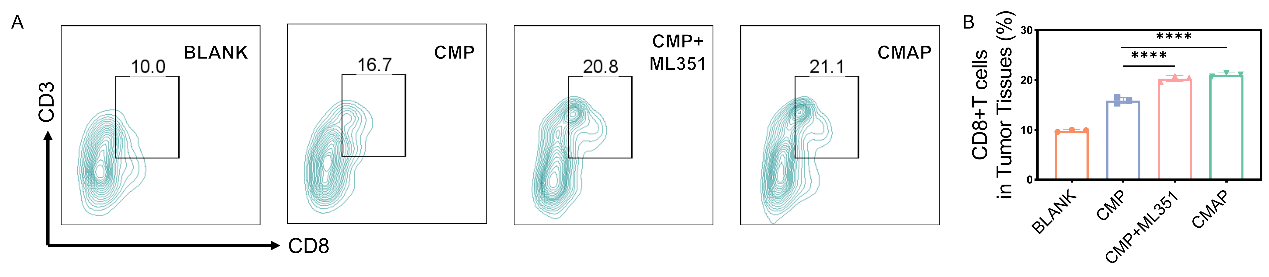


**Figure S22**. (A)Flow cytometry analysis of CD8+ T cells (CD45+CD3+CD8+) in tumor samples extracted from mice after different treatments and (B) statistical analysis. ****p < 0.0001


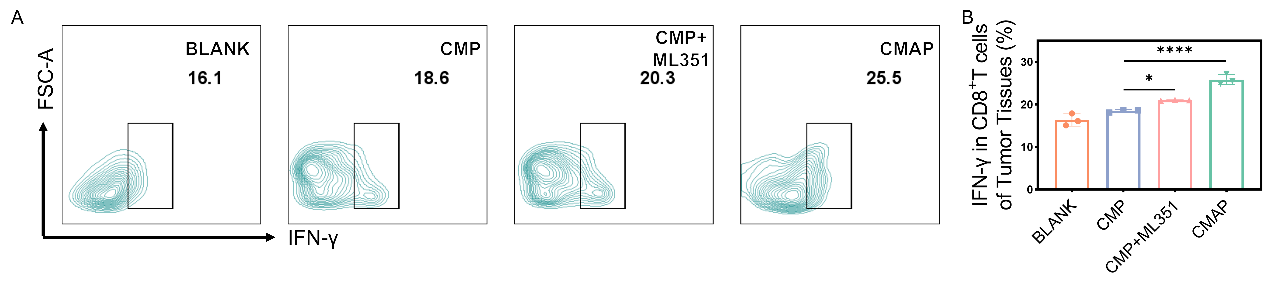


**Figure S23**. (A)Flow cytometry analysis of IFN-γ production in CD8+ T cells (CD45+CD3+CD8+) from tumor samples after different treatments and (B) statistical analysis. *p < 0.05 ****p < 0.0001.

**
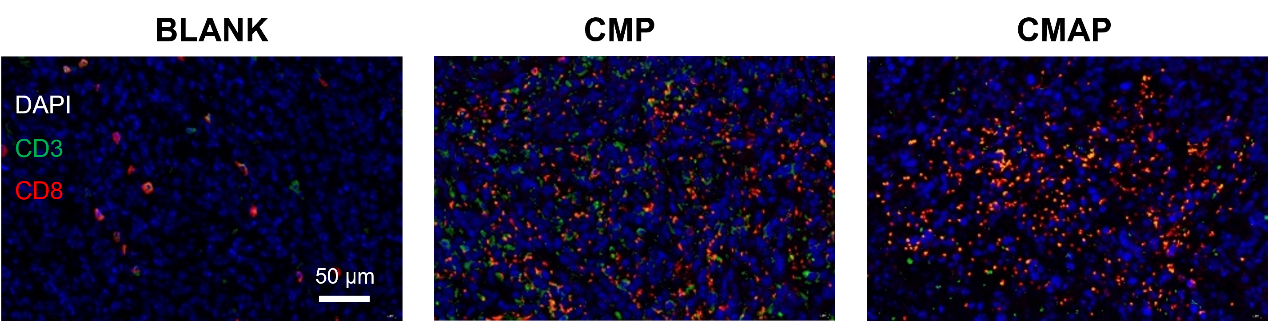
**

**Figure S24.** Immunofluorescence images of tumor sections for CD8+ T cells stained with CD3 (green), CD8 (red), and nuclear (blue). Scale bars: 50 μm.

**
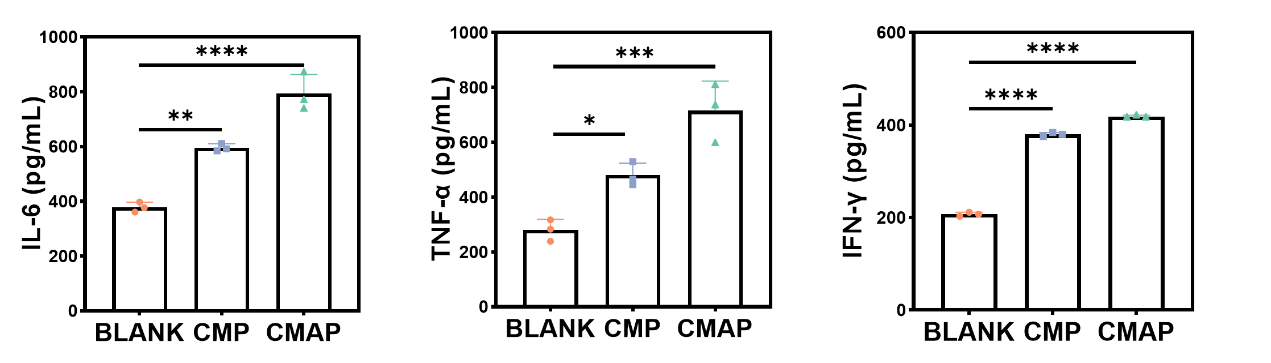
**

**Figure S25.** Cytokine levels of IL-6, TNF-α and IFN-γ in supernatants of tumors derived from treated mice. Data are presented as mean ± SD (n = 3). *p < 0.05, **p < 0.01, ***p < 0.001, ****p < 0.0001.

**
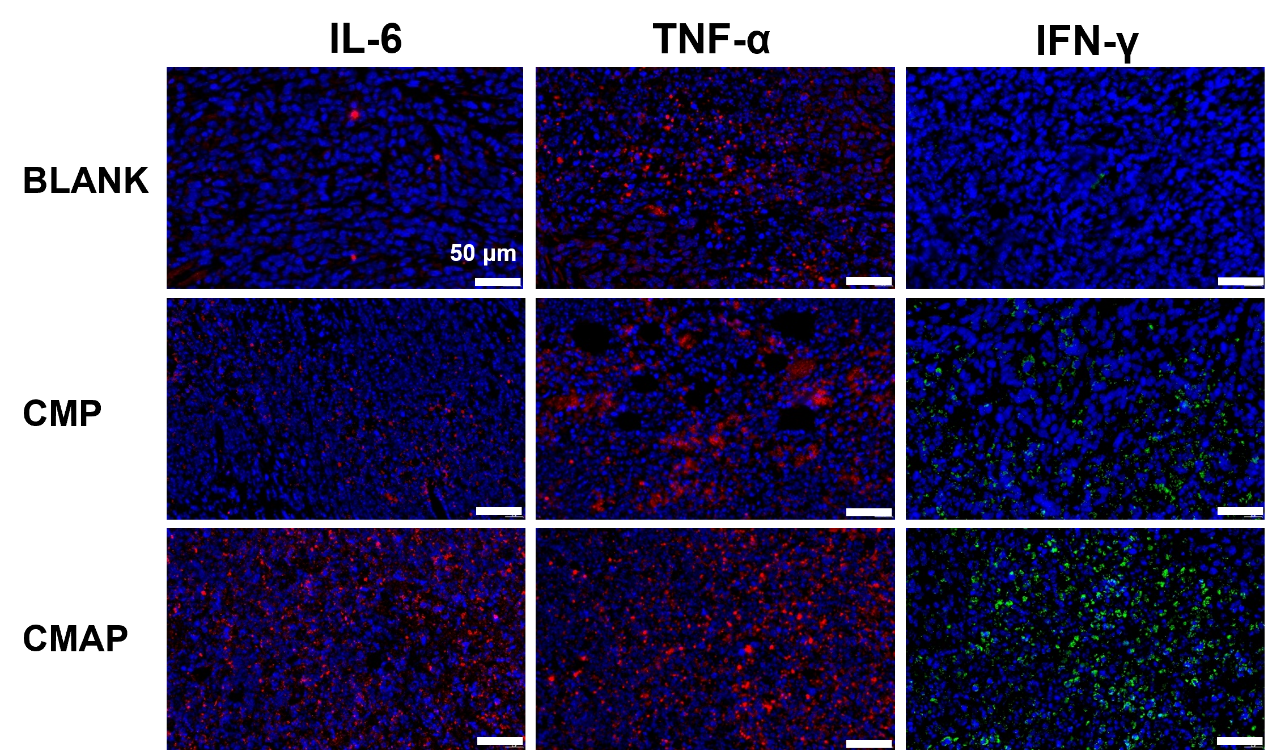
**

**Figure S26.** Immunofluorescence images of tumor sections for IL-6(red), TNF-α(red), IFN-γ(green). Scale bars: 50 μm.
